# Advancing inclusion in sports for students with disability: A mixed-methods study on awareness and perspectives toward adaptive sports

**DOI:** 10.1371/journal.pone.0349033

**Published:** 2026-05-20

**Authors:** Monira I. Aldhahi, Bodor H. Bin sheeha, Noura I. Alothman, Reem M. Basuodan, Reem A. Alabdulwahab

**Affiliations:** 1 Department of Rehabilitation Sciences, College of Health and Rehabilitation Sciences, Princess Nourah bint Abdulrahman University, Riyadh, Saudi Arabia; 2 Department of Health Communication Sciences, College of Health and Rehabilitation Sciences, Princess Nourah bint Abdulrahman University, Riyadh, Saudi Arabia; 3 Department of Teaching and Learning, College of Education and Human Development, Princess Nourah bint Abdulrahman University, Riyadh, Saudi Arabia; ISSEP Kef: Universite de Jendouba Institut Superieur du Sport et de l’Education Physique du Kef, TUNISIA

## Abstract

Adaptive sports promote inclusion, participation, and well-being in individuals with disabilities. However, limited research has examined the knowledge, awareness, and perceptions of adaptive sports, as well as institutional support and barriers within Saudi universities. An explanatory sequential mixed-methods design was employed. A cross-sectional survey (n = 350; 18.29% reported disabilities; 70.57% females; mean age 28 ± 11 years) assessed knowledge, awareness, perceptions, and perceived barriers to adaptive sports among students, faculty, and administrative staff. Survey findings informed purposive sampling for the qualitative phase, which included focus group discussions with faculty and semi-structured interviews with students with disabilities (SWDs) to contextualize quantitative patterns. The qualitative phase included a focus group discussion, and six interviews were conducted virtually using Microsoft Teams. Participants demonstrated uneven knowledge of adaptive sports (mean score: 8/16). While most correctly recognized their role in enhancing physical education and promoting participation through adapted equipment (>83%), but low understanding of classification (19.71%) and healthcare roles (30.57%). Awareness was low across all domains, particularly in training (75.71% unaware) and participation (80.57% unaware), indicating significant gaps in exposure and education. Although most participants recognized the benefits of adaptive sports, they noted the lack of sufficient policies, accessible facilities, promotion, and staff support. Less than 30% reported inclusive programs, and 16.29% perceived equal funding for parasports training. The key barriers included a lack of awareness (43.14%), inaccessible facilities (41.42%), and limited resources (40%). Interviews with students with disabilities and focus groups with faculty and specialists revealed positive experiences, emphasizing personal growth and social inclusion, but also identified challenges in infrastructure, recruitment, and policy. Despite favorable perceptions, substantial gaps persist in knowledge, accessibility, and institutional commitment. Addressing these issues through awareness campaigns, improved facilities, and inclusive policy development is vital for advancing adaptive sports participation and fostering a culture of inclusion in Saudi higher education.

## Introduction

The concept of inclusivity in sports involves adapting sports to accommodate individuals with diverse disabilities [1]. The International Convention on the Rights of Persons with Disabilities emphasizes that individuals with disabilities should have the full right to engage in sporting activities alongside their non-disabled peers in inclusive environments [2]. In recent years, adaptive sports have gained attention as a means of creating accessible environments for individuals with disabilities. In Saudi Arabia, this growing emphasis aligns with the National Vision 2030 agenda, which prioritizes enhancing quality of life, social inclusion, and equal opportunities for all citizens, including persons with disabilities [3].

Research has shown that incorporating adaptive sports programs into educational settings has a significant positive impact on physical and mental well-being and social skills [4–6]. Beyond individual outcomes, adaptive sports programs positively affect the attitudes of students with and without disabilities [6]. In higher education contexts, adaptive sports programs have been shown to expand access to athletic participation for students with disabilities (SWDs) while fostering meaningful interactions between students with and without disabilities, thereby contributing to a more inclusive and supportive campus environment [7]. Participants in such programs frequently reported positive emotional experiences, enhanced self-esteem, and a strong sense of belonging and community.

The success of adaptive sports programs in educational settings is significantly influenced by many factors, including students’ and teachers’ awareness and perceptions. Research indicates that a lack of understanding about adaptive sports among individuals with disabilities, their family members, and community members without disabilities represents a significant barrier to increasing physical activity among individuals with disabilities [8]. This suggests that raising awareness among all groups can lead to higher participation rates and improved integration of adaptive sports into the broader community. Research on Unified Sports has found that students with and without disabilities, as well as coaches, valued the inclusive nature of these programs [9]. Such a positive perception toward inclusion suggests that increasing awareness and fostering supportive attitudes among students and teachers could contribute to the successful implementation of adaptive sports programs.

Despite the growing international evidence base, research examining knowledge, awareness, and perceptions toward adaptive sports in Saudi Arabia remains limited. To our knowledge, only two studies have explored attitudes toward inclusion of SWDs in sports and physical education (PE) in Saudi schools. Salim (2021) examined the attitudes of students aged 10–16 years toward including SWDs in PE classes and found overall neutral attitudes with more positive views among female students, those with disabled family members, less competitive students, and those in schools including peers with disabilities [10]. Alhumaid et al. (2022) investigated attitudes of PE teachers in Saudi schools, and revealed moderately positive attitudes toward inclusion, with female teachers and those with more experience teaching SWDs showing more favorable views [11].

Although these studies provide valuable insights into inclusive practices within school-based PE settings, their findings cannot be directly generalized to higher education or to adaptive sports programs specifically. Consequently, there remains a notable gap in the literature concerning the awareness, knowledge, and perceptions of university students and faculty regarding adaptive sports in Saudi Arabia. Addressing this gap is critical, as universities play a pivotal role in shaping inclusive cultures and promoting lifelong engagement in physical activity. Accordingly, this study aimed to assess the levels of knowledge, awareness, and perceptions regarding adaptive sports among university stakeholders in Saudi Arabia; identify perceived institutional, infrastructural, and social barriers to adaptive sports participation; and use qualitative inquiry to explain and contextualize quantitative findings related to awareness gaps and institutional support. Understanding faculty and students’ perspectives and awareness of these programs is crucial to their successful implementation and development.

To provide an in-depth understanding of these multifaceted issues, this study employed a sequential mixed methods design, combining qualitative and quantitative data collection and analysis. This approach allows for a comprehensive exploration of perceptions while capturing broader patterns across the university population including both individual with and without disability. The study findings will significantly contribute to the efficient planning and execution of adaptive sports programs in Saudi universities, ultimately promoting the rights of SWDs to participate in sports activities throughout their university journey.

## Materials and methods

### Study design.

This study employed an explanatory sequential mixed-methods design. In this design, quantitative data were collected and analyzed first, followed by a qualitative phase to further explain and contextualize the quantitative findings. The mixed-methods approach was selected to provide a comprehensive understanding of awareness, knowledge, perceptions, and institutional barriers related to adaptive sports within Saudi universities. This explanatory sequential design allowed quantitative findings to guide the qualitative phase, enabling a deeper exploration of the issues identified in the survey data [12].

In the first phase, a cross-sectional quantitative survey was conducted to measure levels of knowledge, awareness, perceptions and identified the prevalence of evidence-based barriers and facilitators. Based on prior empirical studies, predefined factors (e.g., accessibility, equipment availability, social support, safety, motivation, and perceived competence) were operationalized into measurable items of barriers and facilitators. The quantitative data were analyzed descriptively to identify overall patterns within the university population, including gaps in awareness, institutional support, and perceived accessibility of adaptive sports programs.

The findings from the quantitative phase informed the qualitative phase, particularly in identifying areas requiring deeper exploration, such as low levels of awareness and perception of institutional support for adaptive sports. These results guided the development of the qualitative interview and focus group discussion (FGD) guides and informed the purposive selection of participant targeting and faculty members who could provide contextual explanations for the observed patterns in the survey data.

The qualitative phase involved semi-structured interviews with SWDs and focus group discussions with faculty members. The first pathway aimed to explore SWDs’ experiences with adaptive sports within Saudi universities and the barriers they encounter. The second pathway examined faculty perspectives on adaptive sports, including factors contributing to limited awareness and institutional support, as well as potential strategies to overcome identified barriers.

The integration of quantitative and qualitative findings occurred at three stages. First, integration occurred at the design stage through the adoption of an explanatory sequential framework. Second, integration occurred at the sampling stage, where survey results identifying low awareness and perceived institutional barriers informed the purposive recruitment of qualitative participants. Finally, integration occurred at the interpretation stage, where qualitative insights were used to contextualize and explain quantitative trends.

### Study procedure & participants

This study adhered to ethical guidelines, including obtaining Institutional Review Board (HAP-01-R-059) approval (IRB Log Number: 23–0876) and collecting informed consent from all participants before their inclusion in the study. The quantitative phase of data collection was conducted over an eight-month period, from January 1, 2024, to August 31, 2024, during which participants were recruited via an online questionnaire. The qualitative phase, comprising all focus group discussions (FGDs) and interviews, were conducted within a single concentrated period during September and October 2024, immediately following the close of the quantitative phase. The overall study period ran from January 1, 2024, to December 31, 2024, encompassing both phases of data collection. Written informed consent was obtained electronically via the questionnaire cover page, which included a clear consent option (agree/disagree) before proceeding to the survey. The cover page and registration link outlined the purpose of the study, the voluntary nature of participation, procedures to ensure confidentiality and anonymity, and the participants’ right to withdraw at any time. Only participants who provided consent were included in the study. Participants were recruited through university mailing lists, student communication platforms, and academic networks across multiple Saudi universities. To optimize response rates during the data collection period, reminder emails were sent every two weeks to non-respondents, encouraging their participation. Upon providing their consent, respondents were immediately granted secure access to the online survey platform. The survey was designed to take approximately 20 minutes to complete.

A non-probability convenience sampling method was used to select individuals willing to share insights relevant to the study objectives. To guide the target sample size, an initial sample size of approximately 384 participants was estimated using a single-proportion formula, n = Z² p(1 − p)/d², with a conservative prevalence of 50%; however, this estimate is based on probability sampling assumptions and is presented as a general reference only. The final sample included 350 participants after excluding incomplete and ineligible responses. Participants were recruited from multiple regions of Saudi Arabia (Eastern, Central, Northern, Southern, and Western) to enhance diversity. However, we acknowledge that geographic diversity does not confer statistical representativeness, as no formal sampling frame or regional quotas were applied.

Using purposive sampling, faculty members were recruited to participate in three medium-sized FGDs, each comprising six faculty members. Recruitment was conducted through the distribution of a registration link for the FGDs and interviews, which was shared with participants who had previously taken part in the quantitative phase of the study. The FGDs included participants from diverse ethnic backgrounds and specialties who were actively engaged in academic or administrative roles. To ensure ethical sensitivity, semi-structured interviews were conducted with six SWDs in each session.

### Data collection methods

#### Quantitative data collection.

The quantitative phase of this study used a structured questionnaire to address six key domains: demographics and personal questions, knowledge of adaptive sports, awareness and perception, strengths and resources, barriers to participation, and SWDs’ perspectives on obstacles to adaptive sports programs in Saudi universities. The items were developed in specifically for this study, as a comprehensive review of the literature identified no single validated instrument that simultaneously assesses knowledge, awareness, and perception of adaptive sports within the Saudi university context. Item development followed a systematic, multi-stage process. First, an extensive literature search was conducted to identify relevant constructs, theoretical frameworks, and item pools related to disability sport awareness and attitudes. Based on this review, an initial pool of items was generated in Arabic, covering respondents’ familiarity with adaptive sports programs, recognition of institutional resources, and beliefs regarding the benefits and importance of adaptive sports for SWDs. The questionnaire structure, item format, and response options were then determined. Items were subsequently reviewed and refined by a multidisciplinary expert panel comprising Sports Medicine specialist, Physiotherapists (both academics and clinicians)— selected to represent the range of stakeholders directly engaged with adaptive sports in Saudi Arabia.

This online survey, comprising six sections, was distributed to students, faculty, and administrative staff at universities in Saudi Arabia using SurveyMonkey® (Palo Alto, CA, USA). The demographic section gathered information on gender, age, education level, university role, and disability. Knowledge of adaptive sports refers to respondents’ factual understanding of the field, including basic concepts, purpose, and eligibility criteria. The items were developed to cover five key domains: (1) definitions and scope of adaptive sports; (2) the role of adapted sports in PE and teaching practice; (3) sport classification systems and frameworks for individuals with disabilities; (4) benefits of adaptive sports for social inclusion, community reintegration, and quality of life; and (5) the role of healthcare and allied professionals in promoting participation. These domains were informed by standard adapted PE textbooks and established literature by Winnick and Porretta [13], to ensure alignment with core concepts in the field. It comprising 16 questions (six true/false and ten multiple-choice). Scores ranged from 0 to 16, with percentages calculated.

Awareness and perception items were developed specifically for this study, as a comprehensive review of the literature did not identify a single validated instrument. The items were adapted from relevant literature, and guidance from previous studies was used to support item construction [8,14–17]. The Awareness and perception section comprised 16 items. The awareness section was assessed using six questions that reflected respondents’ familiarity with adaptive sports and their recognition of available programs, opportunities, and institutional resources within the university context using a structured categorical format (Yes/ No/ I am not aware). The remaining ten items assessed perception using a 5-point Likert scale. The perception represents respondents’ beliefs and interpretations regarding the benefits, importance, and institutional role of adaptive sports, as well as the perceived level of support for SWDs.

To assess the psychometric properties of the newly developed scales, face validity was evaluated by three panel members who assessed the clarity, comprehensibility, and appropriateness of individual items. Content validity was assessed by five panel members who evaluated item relevance, representativeness, and coverage of the intended constructs. Items were revised iteratively in response to panel feedback before being finalized for piloting. The reliability and preliminary structural validity were examined using data from the pilot study (*n* = 30) participants and were excluded from the main study sample. Internal consistency was estimated using Cronbach’s alpha, and principal component factor analysis was conducted to explore the underlying factor structure of each scale. The Knowledge scale demonstrated good internal consistency (α = .878), with six factors retained explaining 80.78% of the total variance. The awareness scale showed acceptable reliability (α = .758), yielding a two-factor solution that accounted for 69.17% of the variance. The perception scale exhibited excellent internal consistency (α = .945), with two factors explaining 84.65% of the total variance: the dominant first factor alone accounted for 67.43%, suggesting a near-unidimensional structure. Full reliability coefficients and factor loadings are reported in the Supplementary File 3.

The strengths and resources section consisted of six items on initiatives, accessibility of adaptive sports facilities, and funding resources within universities. Items in the strengths/resources section were designed using a structured categorical format (Yes/ No/ I am not aware) to assess participants’ perceptions of the availability of resources and institutional support. Barriers to participation among university SWDs were examined through a question with seven selectable options: accessible facilities, awareness, social stigma, financial constraints, limited resources, and other barriers. The final section gathered students’ perspectives on obstacles to establishing adaptive sports programs in Saudi universities, considering personal, social, and environmental factors, and included an open-ended question on barriers and potential solutions. Items sources, all barrier items (both for non-SWDs and SWDs sections) were developed based on an extensive review of the literature on adaptive sports participation, disability inclusion, and environmental and personal barriers to physical activity [18–20]. This section captures the perceived institutional and environmental barriers from the perspective of both SWDs and non-SWDs, particularly faculty and staff who are involved in program planning, administration, and service delivery. Their perspectives are important in identifying potential structural and organizational limitations that may affect the implementation of adaptive sports programs. The survey of the quantitively components were reported in accordance with Checklist for Reporting Results of Internet E-Surveys (CHERRIES) [21].

#### Qualitative data collection.

The qualitative phase, including both FGDs and six interviews, was conducted virtually on the Microsoft Teams platform (version 2,402; Microsoft Corporation; WA, USA) and digitally audio-recorded and transcribed. The FGDs were conducted with each group comprising six participants across the qualitative phase. The FGD and interview questions were derived from the critical analysis of the quantitative findings, in which sections on barriers and facilitators required an in-depth understanding of how to mitigate these factors.

One moderator conducted all the sessions with two note-takers (assistant moderators). All sessions followed a similar flow: beginning with a welcome, clarifying the study’s objective, and establishing ground rules before moving into discussions guided by semi-structured questions, before concluding. Follow-up questions were used if needed to encourage participants to elaborate and provide more nuanced insights into their opinions and experiences. At the conclusion of each session, participants were invited to share additional reflections regarding adaptive sports. All digital audio recordings, transcripts, and informed consent forms were stored electronically on a password-protected computer and were accessible only to the study researchers. To ensure participants’ confidentiality, each participant was assigned a pseudonym, which served as the only identifier on all documents related to them, except for the informed consent form. The qualitative component of this mixed-methods study was reported in accordance with the COREQ (Consolidated Criteria for Reporting Qualitative Research) guidelines [22].

### Data analysis

#### Quantitative analysis.

Survey and demographic data were analyzed using Stata version 16 (StataCorp, College Station, TX, USA). Continuous variables are presented as means and standard deviations (SD), while categorical variables are presented as frequencies and percentages (%), and medians and interquartile ranges (IQR). The analysis focused on descriptive statistics because the primary aim was to provide an overview of knowledge, awareness, perceptions, and perceived barriers across university stakeholders

The threshold for knowledge classification was set at 70%, and scores of 70% or higher were considered adequate, while scores below 70% were considered insufficient. Awareness was assessed using a binary scoring system, in which each item was coded as “Yes,” “No,” or “I am not sure.” Individual responses were summed to generate an overall awareness score. To facilitate interpretation, the total score was converted into a percentage representing the proportion of items answered “Yes.” Based on predetermined thresholds, participants were classified into three categories: high (≥70%), moderate (40–69%), and low or uncertain (≤ 40%) awareness. These thresholds are commonly used in knowledge and awareness assessments to facilitate the interpretation of composite scores in survey-based studies [23–25].

Perception was measured using 10 items rated on a five-point Likert scale ranging from 1 (strongly agree) to 5 (strongly disagree**).** All perception items were positively worded; therefore, lower scores reflected more positive perceptions, whereas higher scores reflected less favorable perceptions. The overall perception score was summarized using the median and interquartile range (IQR) across the 10 items. Perception levels were classified based on the median Likert score as follows: median < 3.0: high (positive) perception; median 3.0–3.9: moderate perception; and median ≥ 4.0: low (negative) perception. This classification was used because agreement with positive perception statements yields lower Likert values (1–2), whereas disagreement yields higher values (4–5).

#### Qualitative analysis.

The qualitative data were analyzed using thematic analysis by two independent researchers following the approach outlined by Braun and Clarke (2006) [26]. The analytical process began with repeated readings of the transcripts to gain familiarity with the data. Segments of text that were relevant to the research questions were then coded and organized into categories. These categories were further refined and clustered into broader themes that captured participants’ perceptions, experiences, and interactions related to adaptive sports.

The development of themes was not linear but progressed through a cyclical process of reflection and refinement. Initial codes were revisited, grouped, and reshaped as new insights emerged, keeping the analysis flexible and grounded in the participants’ accounts. Over time, these themes were consolidated and defined, providing a coherent framework for interpreting the data. This iterative process involved continuously comparing newly coded data with previously analyzed material until no additional insights or themes were generated, ensuring that the analysis reached a thematic formulation.

To enhance rigor, the analyses conducted independently by the two researchers were subsequently merged, and discrepancies were discussed and resolved through consensus to ensure the reliability and credibility of the findings. This step aligns with best-practice recommendations for thematic analysis, which emphasize independent coding followed by a collaborative review to enhance trustworthiness and reduce subjective bias.

Data saturation was assessed iteratively across both the interviews and FGDs. Saturation was considered reached when no new codes or themes emerged across successive sessions and when recurring patterns were consistently confirmed across participants. This point was determined following a review of the transcripts after each session, with the final two interviews and third FGD yielding no additional themes beyond those already identified.

## Results

**Fig 1:**
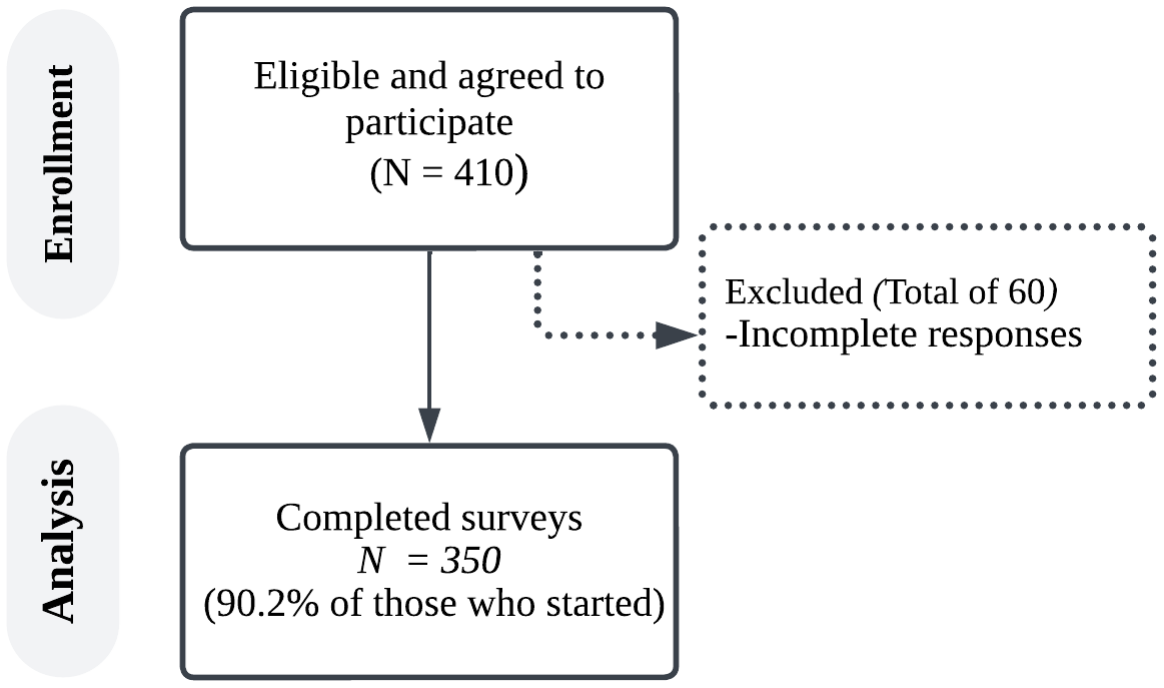
Flow diagram of the study. Flowchart illustrating the study design and participant progression through each stage, including screening for eligibility, allocation, and final sample analyzed.

A total of 410 respondents completed the survey, of whom 60 were excluded from the analysis due to missing data or ineligibility to participate, resulting in a final analytical sample of 350 participants (Fig 1). The majority of respondents (70.57%) were female, and the mean age of participants was 28 ± 11 years. Most of the respondents, students and faculty from various universities in Saudi Arabia, come from different regions, and 18.86% had more than 7 years of experience at their universities. Of those who completed the survey, 64 were SWDs (18.29%). The most commonly reported disability was mobility/physical (65.62%), followed by visual impairment (15.62%), hearing impairment (14.06%), and cognitive disability (4.69%), as shown in Table 1.

**Table 1:** Demographic and Physical characteristics of the participants (N = 350).

Variables	N (%)
Age (mean ±SD, years) *	28 ± 11
Gender, n (%)	247 (70.57)
Female	247 (70.57)
Male	103 (29.43)
Education	
High School Diploma or equivalent	141(40.29)
Associate degree (e.g., A.A., A.S.)	140 (40)
Bachelor’s Degree (e.g., B.A., B.S.)	34 (9.71)
Master’s Degree (e.g., M.A., M.S.)	33 (9.43)
Doctoral Degree (e.g., Ph.D., Ed.D.)	2 (0.57)
Employment (N = 339)	
Student	175 (56.08)
Faculty Member	137 (43.91)
Administrative Employee	27(7.71)
Unemployed	11 (3.14)
Experience at the University	
Less than 1 year	134 (38.29)
1-3 years	95 (27.14)
4-6 years	55 (15.77)
7 + years	66 (18.86)
Diagnosed with disabilities	
No	286 (81.71)
Yes	64 (18.29)
Type of disability (N = 64)	
Physical impairments	42(65.62)
Visual impairments	10 (15.62)
Hearing impairments	9 (14.06)
Cognitive impairments	3 (4.69)
Autism spectrum disorders	0
Region of Saudi Arabia	
Central	100 (28.57)
Northern	85 (24.29)
Eastern	70 (20.00)
Western	55 (15.71)
Southern	40 (11.43)

*Data presented as mean ± SD for data on a ratio scale

Abbreviations: Frequency (N) and percentage (%) for nominal classifications.

### Knowledge about the adaptive sports for SWDs

Table 2 presents participants’ responses to individual question assessing knowledge of adaptive sports across multiple domains. Overall, responses varied considerably across items, indicating uneven understanding.

**Table 2:** Participants’ responses to knowledge-based statements related to adaptive sports across multiple domains.

Items	Response		Domain
	Correct	Incorrect	
	N (%)	N (%)	
Traditional sports have equivalent adapted versions with similar rules for individuals with disabilities.	130 (37.14)	220 (16.57)	Definitions & Scope
Integrating adapted sports into traditional curricula enhances program quality and promotes social interaction among students of all skill levels.	292 (83.14)	58 (16.86)	Physical Education
Adaptive sports enable participation for individuals with disabilities through the use of assistive devices and modified equipment.	291(83.14)	59 (16.86)	Definitions & Scope
Healthcare professionals do not play a role in recommending adaptive sports participation following injury or mobility-related conditions.	107 (30.57)	243 (69.43)	Healthcare Roles
Adaptive sports provide fewer benefits than traditional sports in terms of community reintegration, life satisfaction, employment opportunities, and quality of life.	126 (36.00)	224 (64.00)	Benefits
Individuals who are blind are unable to participate in sports activities.	263 (75.14)	87 (24.86)	Benefits
Certain sports are incorrectly classified as group-based adaptive sports.	173 (49.43)	177 (50.57)	Classification
Certain sports are incorrectly classified as individual adaptive sports.	69 (19.71)	281 (80.29)	Classification
Multiple terms are used interchangeably with “adapted sports.”	192 (54.86)	158(45.14)	Definitions & Scope
Physical education plays an important role in supporting and promoting adaptive sports.	235 (67.14)	115 (32.86)	Physical Education
The key components of sport frameworks for individuals with disabilities are not well understood.	60 (17.14)	290(82.86)	Healthcare Roles
Not all sport settings are correctly identified within adaptive sport frameworks.	118 (33.71)	232 (66.29)	Classification
Effective strategies are required to promote inclusion within general physical education programs.	145 (41.43)	205 (58.57)	Physical Education
Teaching adapted sports requires specific structured steps and instructional approaches.	218 (62.29)	132 (37.71)	Physical Education
Sport classification systems are designed to ensure fair and equitable competition.	175 (50)	175 (50)	Sport Classification & Frameworks
Participation in adaptive sports contributes to improved self-esteem and social inclusion among individuals with disabilities.	223 (63.71)	127 (36.29)	Benefits

A high level of understanding was observed regarding the role of adaptive sports in enhancing physical education, with 83.14% of participants correctly recognizing that integrating adapted sports into traditional curricula can improve program quality and promote social interaction. Similarly, a large majority (83.14%) correctly identified that adaptive sports enable participation through assistive devices and modified equipment. However, misconceptions were evident in several areas. Only 37.14% of participants correctly recognized that many traditional sports have equivalent adapted versions with similar rules. In addition, only 30.57% correctly understood that healthcare professionals play an important role in recommending adaptive sports participation.

Regarding the benefits of adaptive sports, 64.00% of participants correctly recognized that adaptive sports offer comparable benefits to traditional sports. In contrast, 75.14% of participants demonstrated incorrect knowledge regarding the participation of individuals who are blind. Performance on classification and framework-related items was generally low. Only 19.71% of participants correctly identified individual adaptive sports, and 17.14% demonstrated understanding of key components of sport frameworks. Similarly, only 33.71% correctly identified sport settings within adaptive sport frameworks, and responses regarding the purpose of sport classification were evenly distributed (50%).

Approximately 67.14% correctly identified the role of PE in adaptive sports. However, fewer participants (41.43%) correctly identified effective approaches for promoting inclusion in general PE settings and 63.71% of participants correctly recognized that adaptive sports contribute to improved self-esteem and social inclusion, reinforcing awareness of their psychosocial benefits. Overall, participants had a median knowledge score of 9 (IQR: 6–11) and a mean score of 8 out of 16 (SD = 3.67).

### Awareness about the adaptive sports

As presented in Table 3, awareness of adaptive sports was consistently low across all six items. Awareness of adaptive sports was consistently low across all six domains. Unawareness was highest for formal education and training (75.71%) and personal or peer participation (80.57%), followed by knowledge of adaptive sports terminology (67.14%), attendance at events (75.71%), and familiarity with the classification system (68.00%). Uncertainty was generally low across most items, with the notable exception of awareness of university-based adaptive sports programs, where 39.43% of respondents were unsure — nearly matching the “No” response rate (42.29%) for that item.

**Table 3:** Awareness among the respondents related to the adaptive sports.

Items	Response		
**Yes** **N (%)**	**No** **N (%)**	**I am not sure** **N (%)**
Have you heard of the term “adaptive sports” before taking this survey?	65(18.57)	235 (67.14)	50 (14.29)
Have you ever attended an adaptive sports event or competition?	64(18.29)	265 (75.71)	621 (6)
Have you or anyone you know participated in an inclusive adaptive sports or sport activities for individuals with disabilities at the university?	56(16)	282 (96.57)	12 (3.43)
Are there currently any inclusive adaptive sports programs or initiatives offered at your university?	64(18.29)	148 (42.29)	138 (39.43)
Have you ever received any formal education or training, or orientation related to adaptive sports or inclusion?	52(14.86)	265 (75.71)	33 (9.43)
Are you familiar with the classification system used in adaptive sports to ensure fair competition among participants with varying disabilities?	65(18.57)	238 (68)	47 (13.43)

Data presented as Frequency (N) and percentage (%) for nominal classifications

### Perceptions toward adaptive sports

Using predefined perception criteria: a perception score of ≥ 4.0 indicated low perception, 3–3.9 indicated moderate perception, and < 3.0 indicated high perception. Approximately 72.28% of respondents reported median perception scores of 3.0 or below, reflecting high perception. A smaller proportion of participants (7.71%) fell within the moderate perception range. In contrast, 22.0% of respondents reported scores of 4.0 or higher, indicating a low level of perception (Table 4).

**Table 4:** Descriptive Analysis of Perceptions Toward Adaptive Sports Inclusion, Policies, and Resources at the University Across All Participants (N = 350).

Items	Response					Median (IQR)
	Strongly Agree	Agree	Neutral	Disagree	Strongly Disagree	
	N (%)	N (%)	N (%)	N (%)	N (%)	
The increased knowledge about adaptive sports can benefit your university by improving inclusion and participation	160 (45.71)	77 (22)	27 (7.71)	82 (23.43)	4 (1.14)	2 (1-3)
The university plays a significant role in promoting in-depth knowledge and understanding of adaptive sports among students and faculty	140 (40)	91 (26)	30 (8.57)	88 (25.14)	1 (0.29)	2 (1-4)
Adaptive sports programs can have a positive impact on the overall well-being of SWDs	181(51.71)	59 (16.86)	21 (6)	88 (25.14)	1 (0.29)	1 (1-4)
Parasport opportunities enhance the sense of community and belonging among SWDs at your university	172 (49.14)	63 (18)	31 (8.86)	82 (23.43)	2 (0.57)	2 (1-3)
The existing sports facilities for SWDs are accessible	82 (23.43)	55 (15.71)	98 (28)	107 (30.57)	8 (2.29)	3 (2-4)
There are specific policies or guidelines in place at the university to support adaptive sports programs	79 (22.57)	62 (17.71)	103 (29.43)	97 (27.71)	9 (2.57)	3 (2-4)
There are staff or personnel responsible for coordinating adaptive sports at your university	66 (18.86)	52 (14.86)	113 (32.29)	100 (28.57)	19 (5.43)	3 (2-4)
There is sufficient promotion of adaptive sports or sports for SWDs at your university	59 (16.86)	51 (14.57)	100 (28.57)	122 (34.86)	18 (5.14)	3 (2-4)
The inclusivity of sports culture for SWDs in the university is high	53 (15.14)	57 (16.29)	99 (28.29)	128 (36.57)	13 (3.71)	3 (2-4)
SWDs have equitable access to parasport opportunities and resources compared to their peers without disabilities	69 (19.71)	59 (16.86)	87 (24.86)	114 (32.57)	21 (6)	3 (2-4)

Data presented as Frequency (N) and Percentage (%), Interquartile range (IQR)

Most participants agreed or strongly agreed that increased knowledge about adaptive sports can benefit the university by improving inclusion and participation. Similarly, over two-thirds of respondents perceived that adaptive sports programs positively influence the overall well-being of SWDs. In contrast, perceptions of institutional support and infrastructure were notably less favorable. Only 39.1% of respondents agreed that existing sports facilities are accessible to SWDs, while nearly one-third (32.9%) disagreed. Fewer than half of the participants reported awareness of specific policies or guidelines supporting adaptive sports programs (40.3%). Less than one-third of respondents agreed that there are dedicated staff responsible for coordinating adaptive sports (33.7%). Additionally, 40.3% of respondents disagreed regarding the overall inclusivity of the university sports culture for SWDs. Finally, nearly 39% of respondents disagreed that SWDs have access comparable to their peers without disabilities (median = 3, IQR = 2–4).

### Strengths and resources related to adaptive sports

**Fig 2:**
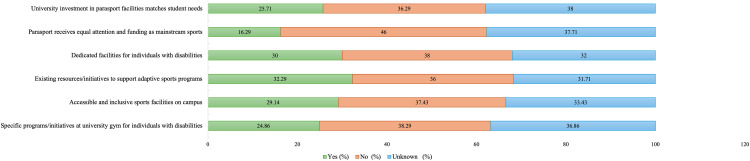
Perceived strengths and resources supporting adaptive sports participation among the overall study sample (N = 350). Stacked bars show percentages of Yes, No, and Unknown responses across institutional support and facility-related items.

An assessment of the strengths and resources within the university related to adaptive sports revealed that most respondents were either unaware of or did not respond to items concerning existing programs, initiatives, designated spaces, or allocated funding for adaptive sports. These findings, summarized in Fig 2, highlight a notable gap in awareness regarding institutional support and investment in adaptive sports at the university level.

Few respondents reported existing adaptive sports initiatives, with fewer than one-quarter identifying specific programs (Fig 2). Similarly, few respondents reported the availability of inclusive facilities or dedicated adaptive sports spaces. Just under one-third (32.29%) identified existing resources or initiatives that could be leveraged for adaptive sports programs. Reports of dedicated facilities or spaces for SWDs were low (30%), as was the perception of equal attention and funding for parasports compared to mainstream sports (16.29%). Only 25.71% of respondents perceived investment in parasport facilities and resources as adequate.

### Barriers to participation in adaptive sports reported by non-SWDs

Insights into barriers affecting participation in adaptive sports were obtained from faculty and students, including those with and without disabilities. Among respondents without disabilities, the most frequently reported barrier to SWDs involvement were lack of awareness, inaccessible facilities, and limited equipment (Table 5).

**Table 5:** Perspectives of University Faculty and Students on Barriers to Participation in Adaptive Sports (N = 350).

Barriers	Frequency	Percentage
Lack of accessible facilities	145	41.42
Social stigma and discrimination	91	26
Lack of awareness and information	151	43.14
Financial constraints	70	20
Limited equipment and resources	140	40

### Barriers to participation in adaptive sports reported by SWDs

Table 6 presents SWDs’ perspectives on the barriers to participating in sports activities at the university. The reported barriers encompassed both personal and social/environmental factors. Within personal factors, the most frequently cited barriers were “*feeling ashamed of one’s disability* (31.25%) and *being dependent on others to be able to exercise* (25%)”. Other personal constraints included disliking participation in sports (15.62%), fatigue (9.38%), and inability to exercise due to the disability itself (7.81%). For social and environmental factors, the most dominant barriers were “*sports possibilities being unknown* (37.50%) and *facilities not sufficiently adjusted* (31.25%)”. Additional factors included financial constraints (9.38%), inadequate adaptation of practice/training (6.25%), limited sports possibilities or qualified supervision in the neighborhood (6.25%), and lack of accessible materials (3.12%). Notably, no respondents reported a lack of acceptance of disabled athletes, lack of peers with disabilities, or inability to find a suitable sport as barriers.

**Table 6:** Perspective of SWDs About the Barriers to Adaptive Sports (N = 64).

Personal factors	Frequency	Percentage
Not being able to exercise because of the disability	5	7.81
I do not like participating in sports	10	15.62
I do not have enough energy/ I am too fatigued to participate in sports	6	9.38
I have an injury that prevents me from participating in sports	1	1.56
Being (too) busy with other activities	3	4.69
Not being comfortable in the presence of other athletes	3	4.69
I am ashamed of my disability	20	31.25
Being dependent on others to be able to exercise	16	25
**Social and environmental factors**	**Frequency**	**Percentage**
Sports possibilities are unknown	24	37.50
Having few sports possibilities in the neighborhood, no/not sufficiently qualified supervision	4	6.25
Facilities not (sufficiently) adjusted	20	31.25
Financial constraints	6	9.38
Transportation	2	3.12
Lack of sufficiently adjusted or available materials.	2	3.12
Practice/Training is not (sufficiently) adapted	4	6.25
Lack of opportunities to exercise with peers.	2	3.13
Disabled athletes are not accepted.	0	0
Lack of fellow athletes with a disability	0	0
Could not find a fitting sport that fits me	0	0

## Qualitative results

### Student’s perspective

Six female SWDs from different universities with different types of disabilities (visual, hearing, and motor impairments) participated in the interview until saturation was reached. (1) experiences of adaptive sports participation and (2) barriers and recommendations for improvement. Illustrative quotes are provided below, with additional quotes presented in Appendix 1(Tables 1 and 2).

### Theme 1: Experiences of adaptive sports participation

Participants described adaptive sports as contributing not only to physical activity but also to personal development and social inclusion. Three interrelated subthemes were identified.

#### Subtheme 1.1: Inclusion and Personal Growth.

Participants consistently reported that involvement in adaptive sports contributed to the development of key soft skills, including time management, self-confidence, discipline, and motivation. Engagement in structured training routines appeared to positively influence daily functioning, with some students reporting improved sleep quality and enhanced concentration.

One participant described how sports participation fostered self-organization and productivity: *“When I got into sports, I felt I found myself in it… my time became organized*.” These accounts suggest that adaptive sports function as catalysts for broader psychosocial development.

Experiences with inclusive training environments, in which students with disabilities were trained alongside non-disabled peers, were described in mixed and evolving terms. Some participants reported initial feelings of embarrassment or self-consciousness; however, others highlighted how inclusive training fostered confidence, motivation, and social connections.

A participant reflected on the positive impact of inclusive practice: “… *the coach wanted to raise their awareness… they trained with their eyes blindfolded. It was a wonderful experience, honestly*.”

#### Subtheme 1.2: Structured Pathways to Participation.

Participation in adaptive sports programs is typically initiated through institutional channels, particularly university support services. Students described a structured process in which sports were selected based on their individual abilities and available resources.

As one participant noted, “*They presented me to the committee… and the committee chose the appropriate sports for me*.” Although this process was generally viewed as supportive, some participants expressed interest in having greater autonomy and exposure to a wider range of sports options.

#### Subtheme 1.3: Training Infrastructure and Coaching Support.

Participants acknowledged the adequacy of existing training facilities and emphasized the importance of qualified coaches in shaping positive training experiences. Access to external ministry-affiliated sports courts was highlighted as an essential resource when university facilities were insufficient. One participant explained, “*The university doesn’t offer goalball… we trained at the Ministry of Sports.*”

### Theme 2: Barriers and Recommendations

Participants’ narratives illustrated that a combination of individual-level concerns, cultural perceptions, and institutional constraints influenced their engagement in adaptive sports. Three interrelated subthemes were identified.

#### Subtheme 2.1: Internalized Stigma and Initial Hesitation.

Many participants described initial fears about participating in sports, including concerns about judgment, competence, and visibility. Family concerns also influenced early participation decisions.

One participant stated, “*I refused at first because I was afraid… my family was also hesitant*.” These findings suggest that psychological barriers may precede participation and highlight the importance of a supportive institutional environment.

#### Subtheme 2.2: Societal Awareness and Constraints.

Participants consistently emphasized low levels of institutional and societal awareness of adaptive sports. Limited media coverage, lack of public recognition, and absence of formal announcements celebrating achievements were perceived as indicators of the marginal status of adaptive sports within university culture. As one participant noted, “*For regular students everything is announced… but for us there is no announcement*.” This lack of visibility was perceived to reinforce stigma, reduce motivation, and limit broader institutional investment in adaptive sports programs.

Structural barriers were repeatedly identified as significant constraints on sustained participation and competitive progression. These included insufficient training periods prior to competition, inadequate transportation arrangements, and limited access to adapted facilities and equipment. Such challenges are viewed as systemic rather than individual, shaping students’ preparedness and performance outcomes. One participant explained, *“…Training outside the university… can discourage students… wasting two hours on transportation*.” Collectively, these findings underscore the importance of infrastructural and organizational support in enabling equitable participation.

#### Subtheme 2.3: Recommendations for Institutional Change.

In response to the identified barriers, participants proposed several recommendations targeting both cultural and structural gaps within adaptive university sports programs. These include enhancing awareness among families, educating them about adaptive sports, and expanding awareness initiatives to reduce stigma. One participant stated, “*I fear that these sports will harm me more than they will benefit me. My family is concerned about me. We need to educate families of people with disabilities so that they can try these sports themselves and gradually introduce them.”* Overall, participants emphasized the need for systemic institutional support rather than isolated initiatives.

### Faculty Perspective

Focus group discussions with faculty members from several Saudi universities identified two themes: (1) the current status of adaptive sports in universities and (2) challenges and opportunities for program development. Additional quotes are presented in the Appendix (Tables 3 and 4).

### Theme 1: The Status Quo of adaptive sports

#### Subtheme 1.1: Perception about Current Status.

This theme captures the current landscape of adaptive sports provision in Saudi universities, revealing a fragmented, largely underdeveloped system constrained by institutional, administrative, and knowledge-based factors. Participants’ accounts indicated that adaptive sports are largely absent at most universities, with implementation limited to a few exceptions. Where provision exists, it is typically confined to students with motor or intellectual disabilities. It is often restricted to recreational rather than competitive sports, suggesting that adaptive sports remain peripheral rather than embedded within institutional athletic structures. One participant stated, “*The university participates in adaptive sports, but there is only one sport in which the university participates. The reason is not the university, but rather the only federation that organizes competitions for people with disabilities, the Athletics Federation*.”

#### Subtheme 1.2: Adaptive sports awareness among university members and decision-makers.

Although some faculty members demonstrated basic awareness of adaptive sports, this awareness was described as uneven and insufficient to drive systematic implementation. As it was stated, “ *I believe there is a tremendous amount of awareness. However, there needs to be guidance or leadership that recognizes this as part of our social responsibility. Universities always have a role in taking responsibility for the less fortunate, whether they are foreigners or people with disabilities.”* Participants emphasized that meaningful progress requires leadership and coordinated initiatives at higher levels of university administration, as well as at the Council of University Affairs, indicating that the marginalization of adaptive sports is closely tied to governance and policy-level prioritization rather than individual attitudes alone.

A lack of awareness of adaptive sports classification systems was identified. Participants reported that a limited understanding of classification principles within university environments may affect the organization of adaptive sports activities and the appropriate inclusion of SWDs. For example, one participant noted, *“No one talks about classification within universities. There is limited knowledge about it, and there are no trained coaches who understand the classification process.”* This lack of awareness may create additional barriers to effective program implementation and inclusive participation.

### Theme 2: Challenges and pathways for advancing adaptive sports

This theme highlights the interconnected challenges that constrain the development of adaptive sports at Saudi universities and provides strategic recommendations to advance equitable access and improve the quality of life for SWDs. Participants identified structural, institutional, and personal barriers that collectively limit the effective implementation and sustainability of adaptive sports initiatives.

#### Subtheme 2.1: Intrinsic and Extrinsic Constraints.

At the structural level, insufficient infrastructure has emerged as a primary constraint. Participants emphasized the lack of appropriate adaptive equipment and suitable transportation, both of which were considered fundamental prerequisites for meaningful participation. These limitations were compounded by a shortage of a specialized workforce, as staff and coaches were often described as lacking adequate training in adaptive sports and in disability-specific needs, underscoring gaps in professional preparation and capacity building. “*The only problem the university may face is the capabilities, facilities, and equipment for these games. Female players are available and have the ability and desire to participate, but the university may need significant support in preparing spaces and playgrounds for people with special needs*.”

Financial constraints also featured prominently, with participants identifying limited funding for training programs, competitions, and awareness initiatives as a significant barrier to program continuity and expansion. Thus, the recruitment mechanism for SWDs was not clearly publicized. Current practices rely predominantly on the Deanship of Admissions and Registration databases, with little evidence of proactive outreach or coordinated recruitment strategies. Consequently, adaptive sports participation remains contingent on administrative records rather than inclusive, institution-wide engagement mechanisms.

Internal communication and coordination were identified as challenges. Participants noted that the effective implementation of adaptive sports requires sustained collaboration among relevant units; however, such collaboration was often fragmented or informal. As one participant stated, “*When we say we’re creating a program, the first thing that must be communicated is communication. Communication between departments is good. I believe there must be communication between the academic departments in the Sports Science Department to qualify and provide cadres capable of modifying sports to be suitable for people with disabilities*.” Beyond institutional factors, participants acknowledged the personal barriers faced by SWDs. Low self-esteem and the influence of unsupportive or stigmatizing external environments were described as limiting participation, illustrating how individual-level challenges are shaped by broader social and institutional contexts rather than personal dispositions alone.

#### Subtheme 2.2: Participant-Driven Recommendations.

In response to these challenges, participants proposed a set of interrelated strategies aimed at systemic change. These included the development of academic and professional training programs to prepare highly skilled adaptive sports coaches, as one participant stated, “*Why didn’t I create a department called Adaptive Sports today, among the departments within the college? Its product specializes only in adaptive sports.”* Furthermore, they indicated the need for improvements in infrastructure and support services to accommodate SWDs, as well as partnerships with disability organizations to enhance community engagement and secure additional funding. Participants also emphasized the importance of establishing an adaptive sports league under the Ministry of Education, viewing it as a mechanism to institutionalize competition, promote equal access, and strengthen national oversight.

## Discussion

These findings highlight several challenges related to the integration and promotion of adaptive sports in universities. A key finding was the uneven knowledge and lack of awareness of adaptive sports in the university community. Misconceptions in the acknowledge were evident in several areas. An important gap in participants’ conceptual understanding of adaptive sports, particularly regarding their structure and the role of healthcare professionals. A notably low proportion of respondents recognized that many traditional sports have adapted equivalents with similar rules, suggesting limited exposure to the fundamental principles of sport adaptation. This misconception may contribute to the perception that adaptive sports are entirely different or less comparable to mainstream sports, potentially reinforcing exclusionary attitudes or reducing support for their integration into existing programs. Furthermore, high response rate demonstrated incorrect knowledge regarding the participation of individuals who are blind, highlighting a substantial gap in understanding inclusivity. Consequently, the performance on items related to classification and frameworks was generally poor, with only a few participants accurately recognizing the role of healthcare professionals in advocating for adaptive sports participation.This highlights a critical disconnect between sports participation and healthcare guidance, despite strong evidence that healthcare providers play a key role in promoting physical activity, rehabilitation, and long-term well-being among individuals with disabilities.

The awareness data further support this, showing that a large proportion of respondents demonstrated low or uncertain awareness, reflecting limited media exposure and insufficient institutional communication. These gaps were also reflected in participants’ perceptions of weaknesses in existing institutional support systems. Participants additionally reported barriers related to facility accessibility, limited dedicated funding for parasports, and fewer specialized programs compared to other university sports programs. The general population identified limited awareness, inaccessible facilities, and insufficient equipment or resources as key barriers. SWDs highlighted cultural and personal challenges, such as feelings of shame and dependence on others, which further intensified these challenges for female SWDs, as well as social and environmental obstacles, including a lack of information about available opportunities and facilities and transportation that is not fully adapted to their needs. The qualitative findings emphasized the previously stated barriers in the quantitative part, as well as other constraints, including insufficient training duration, restricted sports options, a lack of publicized announcements of achievements and dedicated budgets, and a shortage of coaches trained in disability-specific sports modification and classification. Furthermore, family concerns about participation, especially among females, served as a barrier. Addressing these barriers for both faculty and SWDs through awareness campaigns to reduce stigma, improve accessibility, develop policies, and implement targeted investments is critical to fostering a truly inclusive sporting culture in universities. Thus, faculty believe they should have a professional development program focused on adaptive sports and on training qualified coaches. They emphasized the importance of partnerships with disability organizations. Collectively, the findings suggest a strong belief in the value of adaptive sports but point to significant informational, infrastructural, and institutional shortcomings.

### Knowledge, awareness, and perception

Uneven levels of knowledge among study participants were identified, along with a lack of awareness regarding adaptive sports among university communities. Findings show that a high percentage of participants recognized the value of integrating adaptive sports into traditional PE curricula and the impact of this integration on SWDs’ social engagement. This aligns with studies indicating that inclusive and adapted PE environments increase social interaction, participation, and overall program quality [27,28]. Studies have shown that inclusive PE provides meaningful opportunities for SWDs to engage with peers, thereby promoting social inclusion, communication, and a sense of belonging [27,28]. A striking finding was that few participants possessed accurate knowledge of the involvement of blind individuals in adaptive sports. This result shows a considerable gap in participants’ understanding of inclusivity. This finding contrasts with a growing body of literature indicating that individuals with visual impairments can actively and meaningfully participate in sports with appropriate adaptations, such as auditory and tactile cues [29]. Furthermore, research has shown that adapted sports enhance psychological well-being, social inclusion, and overall quality of life among individuals who are blind [30]. Therefore, the misconceptions observed in the current study likely reflect limited exposure to disability-specific knowledge rather than actual limitations in participation.

Lack of awareness within Saudi universities is consistent with broader challenges in the region, where a systematic literature review on sports activities for undergraduate students in Saudi universities indicates varying levels of engagement and awareness [31]. The qualitative findings emphasize a lack of awareness and report that limited media coverage, minimal institutional communication, and the absence of visible adaptive sports programming on campus play significant roles in this limitation. Participants consistently described adaptive sports as “invisible” within university spaces, suggesting that low awareness is not merely an individual deficit but reflects systemic shortcomings in how adaptive sports are positioned and promoted. This convergence between quantitative and qualitative findings aligns with prior research highlighting visibility as a critical driver of awareness and engagement in adaptive sports [32,33]. Studies consistently report a lack of research and low awareness about adaptive sports, particularly for women with disabilities. This gap affects policy, curriculum development, and public understanding [34,35]. By integrating quantitative and qualitative data, this study demonstrates how institutional, infrastructural, and human-capital constraints underpin patterns of low awareness and participation.

### Infrastructure and institutional support barriers

Most respondents highlighted gaps in adaptive sports at universities, citing inaccessible facilities, insufficient supportive policies, and insufficient promotion for SWDs, all of which affect participation. These findings suggest a significant disconnect between the recognized importance of adaptive sports and the perceived reality of institutional commitment and infrastructure. This disconnect is echoed in other studies, which note that, despite known health benefits and mandates for equal opportunity, a comprehensive understanding of how to develop and implement effective adaptive sports programs for SWDs is often lacking [36]. Perceived barriers among participants without disabilities centered on limited access to information and awareness, as well as constraints related to facilities, equipment, and resources. Translating this perception into actionable participation requires addressing the identified barriers. Previous studies have shed light on these factors and reported that a lack of awareness and information, coupled with significant infrastructural limitations such as inaccessible facilities and insufficient equipment and resources, rather than financial constraints, was identified as an impediment to increasing participation in adaptive sports among university students [37,38].

The interviews and FGD data highlighted persistent gaps in facility accessibility and equipment readiness, suggesting a disconnect between institutional intent and operational capacity, which limits the practical implementation of adaptive sports. Divergent views among some faculty members appear to reflect limited exposure to adaptive sports programming for SWDs rather than the absence of infrastructural challenges. Moreover, the lack of dedicated institutional budgets has emerged as a central constraint, restricting universities’ ability to integrate adaptive sports into extracurricular programs. This finding aligns with international evidence showing that insufficient financial investment undermines the sustainability of adaptive sports by affecting facilities, trained personnel, and program continuity [39–41].

### Trainers and classification systems barriers

Qualitative findings indicate that limitations in human capital are central constraints on adaptive sports participation among SWDs. Participants consistently described a shortage of coaches with formal training in sports modification and disability classification, suggesting that workforce capacity shapes participation opportunities. Previous constraints account for the restricted range of sports available to SWDs, which aligns with previous studies demonstrating that inadequately trained personnel undermine access to and social inclusion in adaptive sports contexts [42,43]. In particular, Alharthy et al. (2024) highlighted how insufficiently qualified staff, combined with infrastructural limitations, negatively affect the social integration of persons with disabilities in sports settings [42].

Through the SWDs’ voices, it was revealed that limited trainer qualifications contribute to the narrow selection of sports offers. They reported instances of insufficient planning for SWDs in training schedules and inflexible training sessions that limited their participation in some sports. We believe that this human-capital gap is compounded by a widespread lack of familiarity with international classification systems, which hinders the proper placement of SWDs and limits pathways to regional or global competition. Furthermore, SWDs were not given enough time to train for national and international competitions. This also stems from a lack of institutional support, as reinforced in the FGD. In line with Liu et al.’s meta-synthesis, a significant barrier to participation was that many teachers and other school staff lacked specific training and confidence in adapting physical activity [44]. To improve their experience in adaptive sports, SWDs called for dedicated clubs for each disability to provide specialized training.

### Gender and cultural context barriers

Although the data indicate positive perceptions of the importance and potential benefits of adaptive sports, personal barriers were found to play a significant role. Qualitative data revealed psychological obstacles, such as shame, fear of failure, and perceived dependency, that limit participation, particularly among female SWDs. Notably, 31.25% of participants identified shame as a barrier, suggesting that these experiences extend beyond individual perceptions and may reflect broader socio-cultural influences. In Saudi Arabia and other Arab contexts, disability is often associated with stigma and social sensitivity, which may shape individuals’ willingness to participate in visible activities such as sports. Previous research has highlighted that cultural norms, gender expectations, and societal attitudes toward disability can contribute to internalized feelings of shame and reduced participation [23].

These internal barriers are further reinforced by family resistance, particularly when participation requires travel for sports competitions. This finding may be understood within the Saudi cultural context, where family plays a central role in decision-making, especially for women with disabilities. Studies have shown that some families and communities may discourage or even prohibit women, particularly those with disabilities, from engaging in sports, often due to concerns related to gender norms, safety, and social expectations [19]. Such dynamics may help explain the familial opposition reported by participants in this study. In addition, the transportation challenges reported by participants, including extended travel requirements, may not be solely logistical but also culturally mediated. Cultural expectations regarding women’s mobility and public presence may intensify the burden of travel, particularly when participation involves mixed-gender settings or distant locations. These factors can interact to further constrain opportunities for participation among female SWDs.

This convergence suggests that barriers commonly framed as “personal” are, in part, socially and culturally embedded, aligning with literature that highlights the dynamic interplay among individual, social, and environmental factors in parasports participation [32,33]. Interestingly, the data indicate that factors such as “lack of acceptance of disabled athletes” or “lack of fellow athletes with a disability” were not reported as barriers by SWDs. This finding contrasts with existing literature, which often emphasizes societal stereotyping and discrimination as key constraints. One possible interpretation is that, within the Saudi context, immediate family and culturally mediated constraints may be more salient than broader societal attitudes in shaping participation experiences.

Consistent with previous research, the findings highlight the relative salience of personal versus environmental barriers, as well as the influence of disability type and gender context on participation in adaptive sports [34,35]. This finding also aligns with earlier studies reporting beliefs that sports are “unfeminine” or unsafe, contributing to a “double burden” on women’s participation [19,23]. Additionally, research on Muslim women in disability sports emphasizes issues such as modesty, body coverage, and gender interaction as central considerations, with women-only spaces and culturally appropriate sportswear identified as enabling strategies [36]. Furthermore, the limited visibility and representation of female athletes with disabilities in Saudi Arabia, as noted in previous research, may contribute to their marginalization and reduced access to support and opportunities [23]. This lack of representation can reinforce existing barriers and limit the normalization of participation in adaptive sports.

Overall, the results suggest that personal barriers are deeply shaped by socio-cultural dynamics, particularly for female SWDs. Addressing these barriers requires not only individual-level interventions but also culturally responsive, multi-level strategies that engage families, challenge stigma, and create supportive environments that align with social norms while expanding opportunities for participation.

### Implications for practice and policy

This study has several policy and practice implications for the Ministry of Education (MoE), universities, and Paralympic and other disability-related authorities in Saudi Arabia. Coordinated work across multiple levels is crucial to realizing the objectives of Vision 2030, which emphasize inclusion, quality of life, and increased participation of individuals with disabilities in social and recreational activities via promoting adaptive sports within higher education institutions. Findings showed that very few SWDs had sufficient expertise, as explained by qualitative data indicating a lack of qualified trainers knowledgeable about disability sports classification. Furthermore, data also showed that universities lack accessible facilities, supportive policies, and sufficient promotion for SWDs, which affects SWDs’ participation. The implications of these findings are significant and directly affect the availability of meaningful adaptive sports for SWDs. These findings require establishing policies to ensure the effective application of all measures and the sufficient implementation of adaptive sports.

The MoE is crucial in setting national standards and accountability frameworks, as it is recognized as the main authority responsible for regulating the higher education system.The development of a policy framework and guidelines is imperative to reinforce adaptive sports within higher education institutions, covering minimum requirements for trainers’ qualifications, program delivery, and access to infrastructure. To ensure the availability of accessible facilities, supportive policies, and adequate promotion of SWDs in universities, adaptive sports practices should be incorporated into national higher education quality assurance and certification procedures. Additionally, since qualified teachers are lacking at Saudi universities, pre-service teacher and coach preparation programs can ensure long-term sustainability and enhance capacity at scale by integrating inclusive physical activity modules and adaptive sports. Adaptive physical exercise programs have been demonstrated to increase pre-service PE teachers’ confidence in integrating SWDs [31]. To better prepare future educators to provide inclusive PE environments, it is recommended that these programs be implemented routinely for both instructors and students [32].

National Paralympic and disability authorities play a critical role in addressing human-capital and classification gaps identified in this study. Universities should collaborate with these entities to deliver professional development programs, certification paths, and technical assistance on sports modification, disability classification systems, and athlete development pathways. Strengthening partnerships between universities and national Paralympic organizations can also facilitate talent identification, create clearer progression routes for SWDs, and enhance opportunities for regional and international competition.

Data also shows a disparity between positive perceptions toward adaptive sports and limited knowledge, awareness, and implementation. This gap is further reflected in the quantitative findings, which showed low levels of knowledge and awareness among SWDs. These findings imply the need to institutionalize adaptive sports within campus structures. This calls for universities to incorporate adaptive sports into academic curricula, extracurricular programming, and institutional communication strategies to sustain the visibility and normalization of SWD participation and reduce the feelings of shame identified in the study. Supporting facility adaptation, specialized equipment, and program continuity through a designated budget is crucial to move adaptive sports beyond symbolic inclusion toward meaningful participation.

The identification of shame as a barrier by 31.25% of participants highlights the role of socio-cultural factors in limiting participation. This suggests that attitudinal and psychological factors constrain SWDs’ participation. Accordingly, universities should implement awareness campaigns and peer-support initiatives to reduce stigma and foster inclusive campus environments. Qualitative findings also highlighted transportation barriers, including extended travel times of up to two hours, which may discourage participation despite positive attitudes. This indicates that access is constrained by logistical challenges rather than motivation alone. Universities should therefore consider providing on-campus facilities or transportation support to facilitate equitable participation.

Across all entities, the interaction among personal, environmental, and cultural barriers identified in the study underscores the importance of cohesive strategies [37]. These strategies should integrate raising awareness, elevating trainers’ qualifications, investing in infrastructure, and establishing culturally responsive practices, particularly for female SWDs. Coordinated action among MoE, universities, and National Paralympic and disability authorities is crucial to place adaptive sports as a core component of inclusive higher education and align institutional practice with national inclusion priorities.

Despite the comprehensive nature of this mixed-methods study, several limitations should be acknowledged when interpreting the findings. As participants were recruited from a limited number of Saudi universities, the results may not fully reflect the perspectives of students and staff across the broader higher education landscape. The use of convenience sampling may limit the generalizability of the results to the broader population. Additionally, due to limited sample sizes within specific participant categories, subgroup analyses (e.g., students vs. faculty or SWDs vs. non-SWDs) were not conducted. Therefore, the findings should be interpreted as reflecting aggregated responses rather than differences between distinct groups. Also, the qualitative component relied on voluntary participation in focus groups, which may introduce potential selection bias, as individuals who chose to participate may have had greater interest or engagement in the topic. To mitigate this limitation and enhance representation, invitations were distributed to universities across different regions of Saudi Arabia, encouraging voluntary participation from a diverse academic population. This approach aimed to ensure broader institutional representation and capture varied perspectives across universities. Nevertheless, the possibility of self-selection bias should be considered when interpreting the qualitative findings. Although the qualitative phase included three focus group discussions and six individual interviews, the sample encompassed participants with diverse disability types (visual, hearing, and motor impairments) across different institutional contexts. As such, data saturation could not be fully established within each subgroup, and the findings should be interpreted as exploratory rather than generalizable.

Additionally, the quantitative data were collected over eight months, we acknowledge that we cannot rule out the possibility that adapted sports events, media campaigns, or policy developments occurring during this period may have influenced participant responses. This temporal limitation was not examined and may represent a source of uncontrolled variation in the data. Thus, predominance of female SWDs, and individuals with physical disabilities which may limit the transferability of findings to male students with disabilities. Future research should include more gender-balanced qualitative samples. Finally, while content validity was established through expert review by a panel of faculty members from Saudi universities and reliability and preliminary structural validity was confirmed via a pilot study of 30 participants. Scales were developed specifically for this study and, while demonstrating acceptable to excellent internal consistency and preliminary structural validity, have not yet undergone full psychometric evaluation. We acknowledge that the absence of full psychometric evaluation for this specific population remains a limitation of the study. The instrument has not undergone confirmatory factor analysis or criterion validity testing. Additionally, the absence of reverse-coded perception items may introduce acquiescence bias, and balanced item wording should be considered in future versions of the scale. Future research should prioritise the formal psychometric validation of a standardised knowledge assessment tool for adapted sports in Arabic-speaking university contexts.

### Future direction

Future research should consider longitudinal studies that implement an interventional program

to assess whether awareness campaigns, institutional policies, or adaptive sports programs improve knowledge, attitudes, and participation over time and expand the geographic scope to include a more representative sample of Saudi Arabian universities. Furthermore, research should examine the unparalleled challenges and opportunities, as well as gender disparities, in adaptive sports, with specific attention to ethnic context and social norms that may clearly influence their engagement. This highlights the need for future investigations to include participants with sensory and intellectual disabilities to understand adaptive sports needs across different populations. The future studies should be conducted to examine subgroup differences based on experience and gender disparities across different disabilities to provide a more nuanced understanding of knowledge, awareness, and perceptions among the university community.

## Conclusion

While there is general recognition of the value and potential benefits of adaptive sports within Saudi Arabian universities, the findings indicate a clear need for improved knowledge dissemination, awareness initiatives, and enhanced accessibility of facilities and resources. The results suggest that overall knowledge of adaptive sports among university students and staff remains limited, with many respondents reporting low familiarity with available inclusive sports opportunities. Although participants acknowledged the potential of adaptive sports to promote participation, inclusion, and well-being among SWDs, several barriers were identified. These include inaccessible infrastructure, limited institutional policies, insufficient promotion, and prevailing social stigma. In addition, SWDs reported personal and psychological barriers, such as reduced confidence and dependence on others. Collectively, these findings highlight the need for stronger institutional commitment, including the allocation of dedicated resources, development of supportive policies, targeted awareness campaigns, and integration of inclusive sports concepts within university programs to advance adaptive sports participation in Saudi universities.

## Supporting information

S1 File Quotes for Theme. (PDF)

S2 File Online Survey English Arabic. (PDF)

S3 File Reliability Statistics for Measurement Scales. (PDF)

S4 File CHERRIES Checklist. (PDF)

S5 File COREQ Checklist. (PDF)

S6 Dataset PNE-D-25-55183R1. (XLS)

## Abbreviation:

SWDs: Students with Disabilities
PE: Physical Education
MoE: Ministry of Education
FGD: Focus Group Discussion.
