# Supplementary material for: Advancing inclusion in sports for students with disability: A mixed-methods study on awareness and perspectives toward adaptive sports
Source: PLoS One. 2026 May 20;21(5):e0349033. doi: 10.1371/journal.pone.0349033 (PMC13189293; doi:10.1371/journal.pone.0349033)
Supplement: S1 File — (PDF) [file pone.0349033.s001.pdf]

Supporting information 1: Quotes for Theme

Table 1. Quotes for Theme One Related to Students' Perspectives on Adaptive Sports Experiences

Sub-theme	Illustrative Quotes and Participant ID
**1. Inclusion and Personal Growth**	**"Practicing sports gave me self-confidence; it raised my self-confidence even more, especially since it made me see the same group as myself and a different group. I mean, I say no; these are people—I mean myself—with a disability and the same society and environment. It forced me to step outside of my family's comfort zone and interact with society and the wider world. Exactly, but my social relationships were more open. I only had friends in high school, and I didn't want to get to know anyone at university." R<br>"When I got into sports, I felt I found myself in it. I found that it instilled in me a great deal of confidence in time management. Honestly, my time was very organized. Thank God, I accomplish more. I want to study during my free time so that I can attend training and overcome my laziness. "AS<br>"The sports club is a better place to get to know each other than university. University is all about classes, but the club is not. It is a beautiful thing to observe the university from all angles and from all specializations, as we all benefit from one another. Additionally, I have gotten to know more than one girl at the club as a result. "AM<br>"There were about five healthy students, especially since, thank God, they had a lot of experience. I felt like I was missing a lots. Thank God, their swimming and athleticism were excellent. It made me say, 'No, I'm not here. I'm not that capable.'" R<br>"It was a wonderful experience. At first, we were a team of visually impaired players, playing by ourselves. But after we learned the basics, they insisted on having a team of our own. Then, I suggested to the coach that we train with the rest of the team, not as a team by ourselves, to gain additional experience, benefit from relationships, and gain new ones. This concept represented an improvement in terms of complexity, as it involved engaging in a game with healthy individual. The coach wanted to raise their awareness, and the speed of the performance covering the eyes of the healthy team. They trained with their eyes blindfolded. It was a wonderful experience, honestly." F**
**2.Structured Access and Institutional Pathways to Participation**	**"Frankly, the credit goes to the Support Center for giving me information about these sports." AS, AM, R, F<br>"Frankly, they presented me to the committee, and the committee honestly chose the appropriate sports for me, which were goalball and shot put, and I chose them after that." AS<br>"There was a committee and a coach at the time, and they suggested judo and running. I started running, and honestly, I didn't find myself in it. "I returned to judo because I found it to be thrilling; however, I did not identify with running." F<br>"We've reached the stage where each person knows what's right for us from a health perspective, or from a perspective they know what's suitable for us. For instance, if I have a visual impairment, I am aware of specific**

	**strategies that are effective for me. Therefore, I will experiment with them to determine if they are suitable for me. There's no point in having a committee decide." F**
**3. Training Infrastructure, Resources, and Coaching Support**	**"Yes, there are adequate spaces and assistance. You need help from everywhere, and you find someone to help you, whether with space or people. It's very spacious, thank God. Even the spaces between the equipment when you want to exercise are large. They provide everything for us, thank God." AM.<br>"The regular exercises were convenient and beautiful, but the university doesn't offer goalball. Since the university doesn't have enough space, we trained at the Ministry of Sports. A university location would save time since the trip and return would be long. So, if it were at the university, it would be better." AS**

Table 2. Quotes for Theme Two Related to Students' Perspectives on Barriers and Recommendations

Sub-theme	Illustrative Quotes and Participant ID
**1.Internalized Stigma, Fear, and Initial Resistance to Participation**	**"I was scared of the subject because it was a sport, something new, and I wasn't sure I could do it." I was among those who refused. I was honestly hounded; I mean, they tried to do it with me. Like other students, I refused and feared we couldn't play sports. As a new idea with no information, the family may have rejected it, as did I. It was a daunting topic, necessitating travel, and my family was initially hesitant. However, after consulting with the center and providing comprehensive explanations, they ultimately agreed". R<br>"The truth is, I was watching the girls who were with me. One of them neglected to participate from the outset, and her mother consistently with her due to her apprehension. She consistently voiced her fear, and it is important to remember that anxiety is a natural emotion for new experiences. Sometimes she's afraid that she'll be hurt; she's afraid before something happens. She is merely apprehensive, and we are grateful that the coach rescued her from this situation. As they provided us with a coach with a disability who had achieved international success, we were inspired by her experience. "And we continue as it is, thank God." AS<br>"Yes, it is true that some of them, I mean, at the beginning, I was apprehensive about how I would throw and not use my other hand, and how I would do it. Sometimes, if I got nervous or pulled my hand, I might shake, so I used to say, "How, how can I, and how can I have one hand play and the other not?" However, I am grateful; in all honesty, the trainer did not disappoint, and the experience was entirely different than I anticipated." AM<br>"And some of them also tell you, "Study; I don't want this sport, for example; it tires me out from my studies and takes up my time." Consequently, I observe many individuals who have withdrawn from the club due to this. I don't want to focus on my studies." F**
**2.Societal Awareness and Constraints**	**"I see girls who are regular students entering a sport where there is an announcement of the winner, an announcement of the students participating and winning, an announcement of everything for everyone. But for us people with disabilities, there is no announcement. They don't advertise, and this is annoying." R<br>"There is a lack of awareness. There is no widespread awareness, for example, of an international day for this. There isn't one. I mean, if there's a new sport, there isn't anyone you see practicing it. You wouldn't know." AM<br>"When she starts studying, they teach her and give her news about it. For example, they spread the word. This means that more bodies are available to all members of the teaching staff, and the students know before they reach the final levels." F<br>"Awareness is low because they don't train all year round. It should be done year-round like other games so they can prepare for any team and game." AS<br>"The student gives an example from the time they register at the university, which gives them admission, for example, time to practice certain sports." F**

	**"I see that transportation is linked to the same thing. I am unable to dedicate a significant amount of time to training due to the fact that I lack transportation to reach the location after that time. Training outside the university, for example, is a bit of a different route. This can discourage students from attending because they have time to study, making it difficult for them to get there and back, wasting two hours or an hour and a half on transportation. It's difficult." AM<br>"He tells you, 'I need transportation. I need certain things to practice sports, and I can't afford them.'" AS<br>"I went to a club for people with disabilities here in a city other than Riyadh. The club isn't interested in judo. The most important thing is that it's called a club for people with disabilities, but there's no interest in the quality of training. This is truly unfortunate." F**
**3.Participant-Driven Recommendations for Institutional Change**	**"There should be no limitations in the available sports. For each disability, there is a sport that suits it and a sport that doesn't, especially if they are targeting multiple disabilities, such as motor or auditory disabilities. Each disability has a sport that suits it. This is very, very important. The second thing is to play sports a fundamental element for them and to have a special university election to represent the university in tournaments." R<br>"Encouraging students is crucial, as individuals with disabilities often harbor a significant fear of participating in sports and attempting new activities." This is especially true for me with a disability. I fear that these sports will harm me more than they will benefit me. My family is concerned about me. We need to educate families of people with disabilities so that they can try these sports themselves and gradually introduce them." In summary, it's important to enter, enjoy, and simply give it a try. If you enjoy it or find the passion, continue, and if not, that's it." AM<br>"There is a need for trainers, specifically those who specialize in working with individuals with disabilities, but also for trainers who are self-developing and have experience dealing with these individuals." From my experience, the trainer wasn't specializing in people with disabilities. She was a very ordinary trainer, and she trained the visually impaired team and the regular team. However, she listened to the players, what they needed in training, and so on. During training, we would tell her to do this and that, and she was very cooperative. In other words, from that perspective, I feel that it's not difficult for the trainer himself to accept training a person with a disability." F<br>"Increasing media coverage of tournaments, as this teaches people about adaptive sports and increases participation." In university marketing and media, the support office informs students before their first day of university of the competitions and medals the university has won or can participate in to motivate them to participate." AS**

Table 3. Quotes for Theme One Related to Faculty Perspectives on The Status Quo of Adaptive Sports in Saudi Universities

Sub-theme	Illustrative Quotes and Participant ID
**1.Perception about current status**	**"A while ago, the Special Olympics, related to sports for people with intellectual disabilities, was present and active." 1-S-J<br>"The university participates in adaptive sports, but there is only one sport in which the university participates. The reason is not the university, but rather the only federation that organizes competitions for people with disabilities, the Athletics Federation." 1-S-H<br>"We have students with special needs. There are academic programs in sports. They participate regularly and practice sports outside the university. For example, there are students at Jeddah University who don't train on campus." 1-S-J<br>"Every year, there's participation in the Disabled People's Championship. Not only that, we also conduct an annual evaluation process. It's a comprehensive performance assessment, where we assess our current situation and develop a development plan for the following year. This is what happens every year, by the way. And not only that, we also currently do something called recreational, recreational, and active sports for people with disabilities. Thankfully, this is part of the plan because we've been able to create a new culture in recreational sports for all students and people with disabilities. I can change their attitudes. I can stimulate their talents at that moment to direct them toward this sport and bring them something new. Because I have so many sports." 2-S-JA**

**2.Adaptive sports awareness among university members and decision-makers**	**"For faculty members, awareness exists, as do administrators (the agency for student activities). Specifically, they are able to communicate, interact, and understand the topic, as do faculty members. Students are ready, but they are learning. They are ready, but what can we teach them?" 1-S-H**<br>**"Initiatives from the university administration, as far as I know, no." 1-S-H**<br>**"This is due to the existence of a special education classification, which outlines specific requirements." The focus is on the mental aspect, specifically intellectual disabilities. But we are talking about physical, visual, and hearing disabilities. They all have specific requirements. For example, we talk about hearing or visual disabilities, which require equipment and devices for that. Universities have not delved much into the topic because it requires details, support, and a deep understanding of the requirements." 1-M-N**<br>**"It's not just a matter of awareness. I believe there is a tremendous deal of awareness. They need either guidance or leadership that feels this is part of our social responsibility. Because in university education, universities always have a role in taking responsibility for the less fortunate, whether foreigners or people with disabilities." 1-M-N**<br>**"Every four years, we win the Olympics and Paralympics. I mean, awareness exists at the societal level, but the university must embrace this. It's our responsibility to do it. I think that's the problem." 1-M-N**<br>**"Universities exist, but the nature of academic work doesn't wait for action from above; it comes from below, with a department council adopting it. Therefore, awareness comes primarily from faculty members." 1-M-N**<br>**"The problem is that awareness doesn't translate into action. That's the problem. Just empathizing with them." 1-M-N**<br>**"There is essentially a major deficiency in understanding the characteristics of people with disabilities, both at university and outside of university in general, apart from adaptation, regarding the adaptation of some curricula or sports. There is a very high resistance to adaptation, but we may still isolate them. There is still a lack of awareness of the meaning of integration and the meaning of adapting the environment and activities to include everyone." 2-A-JA**<br>**"I think everyone thinks they understand, and everyone believes that. Then, at the end of the discussion, they are called "sound" and "unsound." 2-A-JA**<br>**"Adaptive sports are adapting the nature of the sport to the nature of the disability itself, meaning adapting existing sports based on the nature of the disability." 2-S-JA**<br>**"The one who integrates becomes more open with people. You sense that the student is even positive in their interactions. That is, even if we talk about soft skills, they have acquired more of them. Beyond soft skills, you sense a difference in their integration with different**

	students. They are more integrated into society, and their academics are better." 2-S-JA<br>"This depends on the coach. The university itself has the willingness and administrative capacity to bring together students and players who are willing and ready. Coaches understand the disability, the game, and whether it's possible to lose. He's the one who ultimately goes through the selection process or the presentation of the idea itself. After that, he goes through the stage of determining whether the player is suitable or not." 1-S-H<br>"No one talks about classification, for example, within universities. This is part of the problem because there was no coach or knowledge of the classification issue." 1-M-N<br>"It's natural that sports, in general, have a positive impact on the individual, whether they have a disability or not. It impacts the development of skills and confidence and even protects against depression. There is no doubt that this positively influences academic and social skills, social integration, and a sense of belonging within the system. It certainly has a positive impact . Simply feeling valued in this place and finding comfort in practicing what you love gives you a sense of belonging to the system." 2-A-JA<br>"Yes, I train them in adaptive sports. I have worked for many years with people with special needs. My brothers are deaf, and I have worked with the blind for many years, both at university and outside of university." 2-A-JA<br>"There is no personal experience. The classification is based on how many you have, how many you are required to compete in, and how many championships you compete in. What are the available championships, how many representatives do you have, and what is your previous experience? It all depends on you. The same applies to utilizing members who have the ability to train this group, whether through recruitment or areas that are far from the center, meaning it is difficult to find the right person, unlike areas in big cities." 2-A-JA<br>If the sport is suitable for the student's disability, they may enter multiple rounds. Alternatively, we could consider an assessment process instead of a test. We give it to the student. Yes. For example, how can we get him or her to enter with me? As I told you a while ago, there are many adaptive sports. The student may, for example, not have an aptitude for a particular sport or any of them." 2-S-JA

Table 4. Quotes for Theme Two Related to Faculty Perspectives on Challenges and Pathways for Advancing Adaptive Sports

Sub-theme	Illustrative Quotes and Participant ID
1.Intrinsic and Extrinsic constrains	"The only problem the university may face is the capabilities, facilities, and equipment for these games. Female players are available and have the ability and desire to participate, but the university may need significant support in preparing spaces and playgrounds for people with special needs." 1-S-H<br>"The space exists but is not designated for these games, and the facilities themselves are not adequately equipped." 1-S-J<br>1-M-N "There are significant costs, and there are equipment costs. The stadium is not equipped inside, and the restrooms are not equipped."<br>"Athletics requires a wheelchair for people with special needs, which is very expensive because it has specific international specifications." 1-M-N<br>"We need a special car equipped and air-conditioned for people with disabilities." 1-M-N<br>The university receives financial support for its involvement in implementing such programs. All universities want to compete with each other. If they are strengthened, leadership support is already available. Competition between universities in implementing such initiatives needs to be strengthened and accelerated. There is a pressing need for operationalization. Yes, operationalization must be done." 2-A-JA<br>"When we say we're creating a program, the first thing that must be communicated is communication. Communication between departments is good. I'm in the Special Education Department. From my perspective, I'm an expert in assessment, providing appropriate treatment, and reducing unwanted behavior. When talking about a disability coach, the College of Sports Sciences and its departments come in. Therefore, I believe there must be communication between the academic departments in the Sports Science Department to qualify and provide cadres capable of modifying sports to be suitable for people with disabilities." 1-S-J<br>"A second aspect of awareness: Community awareness of the importance of sports. You know from many studies that sports are for everyone, not just disabled people. Sports have a positive impact on various issues, including social inclusion and so on, which is something that people with special needs suffer from greatly. So, it is important to raise awareness among families and relatives of people with special needs. I also see this as an obstacle. I also perceive potential financial barriers. People with disabilities might go to special education academies in the community, which are expensive. This is where the role of universities and social

	**responsibility in universities comes in. This is one of the goals of Vision 2030, that the public and private sectors serve the community. This is where the role of universities comes in, establishing programs, of course, according to specific standards, to meet their needs." 1-S-J<br>"There must be an infrastructure that provides comprehensive access for people with special needs." 1-S-J<br>And every club employee can regulate and make the environment accessible without any connection to the issue... He may try hard; he may succeed, but it is a curriculum that exists in universities." 1-S-J<br>"Social aspects remain." The lack of participation from students, family, and friends remains a significant issue. I believe that currently, most of us have not encountered a significant problem due to the intellectual development in society to practice sports at all levels. The second need is facilities and equipment, which I emphasize. The third need is to provide specialists or specialized people in all fields and in all games so that they can guide players, both male and female, more accurately and more consciously... in a very conscious and specialized manner to practice these activities. Of course, the most crucial requirement is financial support, which plays a significant role in all material matters. And frankly, I feel that the biggest problem currently is that we provide the equipment, facilities, and specialized people. All of this means that if it were available, I believe practicing these activities would not be a problem. 1-S-H<br>"Universities are welcoming, but everyone is floundering, and there is no team. There is a voice that emerges as a member of a committee out of eight or seven members, and thus they argue here and argue there and argue there, and the obstacles increase more than the facilities. They appear, and they remember all the obstacles. We are already suffering from the obstacles we have, so how can we bring in and add a burden? Administrations typically avoid disrupting existing conditions. 2-A-JA<br>"I think there must be training; there must be preparation programs, right? Yes, pre-qualification programs, regardless of experience. There are certainly beneficial international practices, laws, and previous experiences. Training on these determines the quality of institutional work." 2-A-JA<br>"I think this obstacle is that it's one of hundreds of large files. Number two, no model says, 'This is it,' for example, 'implement it,' so we can follow it. Whereas if an operational model were sent to help run this sport at the university, I think this would greatly assist in implementing the programs." 2-A-JA<br>"The nature of disability itself creates psychological factors that make them embarrassed to be exposed to situations that involve disappointment, for example. These issues require greater awareness and widespread dissemination of. The programs**

	**themselves, directed by universities or the Ministry itself, the Ministry of Higher Education and Activities, should be urging them to conduct awareness programs on a huge scale because it is likely that we are facing the fact that many girls do not want to participate simply because they are embarrassed." 1-S-H**<br>**"The psychological or self-esteem aspect is definitely present when seeking the help of specialists." 1-M-N**<br>**By breaking the barrier between us, I can help the student feel connected to their environment. That is, if I organize a tournament within the university, I must integrate this student from this category if the tournament or sport allows their participation. As long as the current tournament, competition, sports activity, or recreational activity, whatever its name or program, is considered to be for people with disabilities, we must implement it. I must integrate them so that they do not ask, 'Why am I here?' By doing this, they integrate into the social fabric within the university environment." 2-S-JA**<br>**"Communication skills are very important. This is one of the most important skills we lack in students with disabilities or other groups. The student often feels isolated in interactions. This is true. Over the past three years, the students in the tournament have changed, improved, and become proactive. For instance, they have shown remarkable improvements in teamwork within the sports environment. In the participation, we found that the student who comes to us—thank God—is the one who wants to come. Indeed, he is the one who initiates the initiative. But consequently, at one point, this category actually exists in universities, but it's waiting for someone to provoke it— someone who will go along with it and know how to tell them about it so that he can recruit them into the field. 2-S-JA**<br>**"That means it stems from the individual himself or the student. Alternatively, the student may not explicitly state the reason for the rejection. But his only response is, "No, I don't want to participate." However, there are instances when a student may choose to refrain from participating due to personal reasons. That's true. Additionally, his aversion to the external community may be the cause. 2-S-JA**<br>**"And in some cases, it stems from the family because it wasn't just about practice; it was about travel." 2-S-JA**

Sub-theme	Illustrative Quotes and Participant ID
**2.Participant-Driven Recommendations**	**"The first thing is to establish academic programs focused on adaptive sports for people with special needs, thus providing specialists with experience in modifying sports." 1-S-J<br>"Why didn't I create a department called Adaptive Sports today among the departments within the college? Its product specializes only in adaptive sports." 2-S-JA<br>"The presence of physical education or a physical education trainer dedicated to training people with special needs." 1-S-J<br>"Cooperation between academic programs and clubs, as academic programs help establish policies, for example, and work mechanisms." 1-S-J<br>There is a need to support the Paralympic Disabled Federation. 1-S-H<br>"Federations for people with disabilities need to focus on university participation and provide competitions specifically for them." 1-S-H<br>"Support is not just a stadium. Of course, it also includes space, equipment, and trainers. Yes, space, equipment, and personnel, as well as individual training at the player level." 1-M-N<br>"There's a shortage of qualified personnel in this field, particularly in sports activities." Yes, there is a shortage for one reason: today, because I work with this group, not because I specialize in physical education, I will be able to deal with this group. To emerge as a creative and productive individual from this group, I must have specialists to deal with this group and be aware of the problem." 2-S-JA<br>"Competency development programs for working with people with disabilities, whether in the sports field or the university environment in general. Of course, we're talking about adaptive sports. Even I, who work at a sports station in this environment or with this group, must develop a program for qualified and specialized trainers and staff for this group." 2-S-JA<br>"Also, I believe the Authority for Persons with Disabilities has done a fantastic job in employing people with disabilities. It may be missing the next step, which is to pressure universities to engage in recreational and sports activities for people with disabilities." 1-M-N<br>"I believe that policies and capabilities are crucial." We also benefit from legislative bodies such as the Authority for Persons with Disabilities. The Authority for Persons with Disabilities plays a significant role in employment and accessibility, as they have begun to publish the number of job openings at Saudi**

	**universities annually. Universities have become more interested in this issue and consider it part of their classification and accreditation." 1-M-N<br>"Significant psychological and social support for this group." 1-S-H<br>"Raising awareness among decision-makers and faculty members." 2-S-JA<br>"Universities don't move unless there is legislation for university sports that comes from above. There wasn't a sports federation for university sports in general. To some extent, there was pressure, and it became necessary for us to participate and have international membership. Now, there is a University Sports Federation. I believe that universities, or at least one of them, require specialists to take the lead and exert pressure on the University Sports Federation, given that the federation is already established. It should add special needs sports to the sports it sponsors, and the needs should be organized and sponsored. An annual competition should be held, such as the Paralympic Games, with international participation. For instance, we should collaborate with Gulf and Arab universities in sports such as football, basketball, and volleyball. 1-M-N<br>"We should establish a league, association, or federation for this sport, such as the Disability Sports Association in the Riyadh region, so that it falls under its umbrella. Alternatively, we could call it a league, allowing us to use any name we choose." It could bring them together. Frankly, it's an excellent idea because not all universities have the capabilities, especially private universities, which are not well-prepared." This initiative has the potential to foster cooperation. 1-M-N<br>"The biggest boost is its inclusion in the annual evaluation of the Deanship of Student Affairs at universities, as well as in the evaluations issued by the Sports Authority. The Sports Authority evaluates sports activities at universities with annual points, including programs offered, whether hosting or participating with people with disabilities in these activities. Setting clear criteria (benchmarks) that include their integration will strengthen universities and encourage them to adapt to this path. In addition, there is a clear program sent to universities. Universities compete to collect these points (2-A-JA). Yes, the universities' index in the Saudi Federation for University Sports has a performance indicator that it evaluates universities against, currently and for years. Among these indicators is a field called internal championships, which includes a clause specifically for sports for people with disabilities. This must be implemented within the university." 2-S-JA<br>Furthermore, if a model is proposed, they can adhere to it flawlessly. If there is one model, one model, two models, or three models, then two or three models are guiding. Hopefully, there**

	**won't be significant opposition. But the proposal without guiding models makes the task difficult for them." 2-A-JA<br>"If you made a calendar and included them in it, I think this would enhance it. Enhance it even more. Yes, a programming calendar, and they are among the targeted groups in it. I think, for example, there are large spaces here— working here, working here, working here. I think this would enhance integration and encourage them to come. The entire university is here, not just people with disabilities. Therefore, when you capture a photo and share it on Twitter, please refrain from referring to it as "sports for people with disabilities." So, the university offers inclusive sports programs, this way and that, and they are present in this space." 2-A-JA<br>"Expand the percentage of participation in the sports diversity itself, especially adaptive sports, and raise awareness of it among the group of students within the university. What matters most is their health and well-being, so I focus on integration and recreational sports, not championships. I started with recreational sports exactly like this. In the analogy, I say when someone teaches someone how to swim, they tell him to go down and swim once. No, I get lost. He has a fear of water, so I can expand the reach of the existing base within the university." 2-S-JA<br>"Communicating with associations, especially those in the sector that support people with disabilities in general, means communicating with them, establishing relationships and partnerships with them, and working with universities to promote the issue." We receive charitable donations, but there is a difference between charity and marketing and business. This is one of the problems, and many places around the world deal with it because institutions must support them socially. That is why several associations were established. There are associations related to disability, and I believe universities should communicate with them more and give them a platform. For example, an association in Riyadh and the Paralympic Disabled Sports Federation would welcome anyone who wants to support them." 1-M-N<br>"Hosting tournaments for disability among universities, similar to other sports." 1-M-N<br>"To be frank, I have never been presented with this subject before, nor has it been addressed in the field." 2-S-JA**
