# Supplementary material for: Advancing inclusion in sports for students with disability: A mixed-methods study on awareness and perspectives toward adaptive sports
Source: PLoS One. 2026 May 20;21(5):e0349033. doi: 10.1371/journal.pone.0349033 (PMC13189293; doi:10.1371/journal.pone.0349033)
Supplement: S2 File — (PDF) [file pone.0349033.s002.pdf]

Supporting information

Faculty and Students Perspectives and Awareness toward Inclusive Adaptive Sports on Saudi Universities: Mixed Method Design

Cover Page

Faculty and Students Perspectives and Awareness toward Inclusive Adaptive Sports on Saudi Universities: Mixed Method Design

You are invited to voluntarily participate in a scientific survey for research study that aims to identify potential barriers and challenges that hinder the participation of students with disabilities in sports activities and to assess the awareness and attitudes of both students and faculty members regarding the importance and benefits of inclusive adaptive sports in the Saudi universities.
This research study seeks to enhance our understanding of the experiences and perceptions of individuals in the university community regarding adaptive sports and inclusivity.

Participating in this survey carries no financial benefits, and there are no associated risks. Your generous participation will contribute to scientific knowledge and inform policy decisions. Your participation will remain completely anonymous. Only the study team will have access to the information you provided. By voluntarily participating in this research, you are giving the researcher permission to use your information for the purposes of searching.

You will be asked to fill out the questionnaire, which takes from 15-20 minutes. Your voluntary participation in this survey is greatly appreciated and will be instrumental in advancing our understanding of inclusive sports at Saudi universities. Your insights can contribute to making positive changes and enhancing inclusivity in our community.
You can get more information by contacting the research team via e-mail (Mialdhahi@pnu.edu.sa) and any questions you may have will be answered. If you have questions concerning your rights as a research subject, you may call the PNU Institutional Review Board office at 288-9999 ext. 26913.

We value and appreciate your participation in this research.

Do you give your consent to participate in this survey?

- ☐ Yes
- ☐ No

Section 1: Demographics and Personal Questions

What is your gender?

□ Male
□ Female

What is your Age? -------

Please indicate the highest level of education you completed:

- ☐ High School Diploma or equivalent
- ☐ Associate degree (e.g., A.A., A.S.)
- ☐ Bachelor's Degree (e.g., B.A., B.S.)
- ☐ Master's Degree (e.g., M.A., M.S.)
- ☐ Doctoral Degree (e.g., Ph.D., Ed.D.)
- ☐ None of the above
- ☐ Other: ……..

What is your current role at the university?

- ☐ Student
- ☐ Faculty Member
- ☐ Administrative member
- ☐ Other

How many years have you been associated with the university?

- ☐ Less than 1 year
- ☐ 1-3 years
- ☐ 4-6 years
- ☐ 7+ years

What is the name of your university?

- ☐ Al Baha University
- ☐ Al Jouf University
- ☐ Al Yamamah University
- ☐ Alfaisal University
- ☐ Al-Imam Muhammad Ibn Saud Islamic University
- ☐ Dar Al Uloom University
- ☐ Dar Al-Hekma University
- ☐ Effat University
- ☐ Fahad Bin Sultan University
- ☐ Imam Abdulrahman Bin Faisal University
- ☐ Institute of Public Administration, Saudi Arabia
- ☐ Islamic University of Madinah
- ☐ Jazan University
- ☐ King AbdulAziz University
- ☐ King Abdullah University of Science and Technology
- ☐ King Fahd University of Petroleum and Minerals
- ☐ King Faisal University
- ☐ King Khalid University
- ☐ King Saud bin Abdulaziz University for Health Sciences
- ☐ King Saud University
- ☐ Majmaah University
- ☐ Najran University
- ☐ Northern Borders University

- ☐ Prince Mohammad Bin Fahd University
- ☐ Prince Sattam Bin Abdulaziz University
- ☐ Prince Sultan University
- ☐ Princess Nourah bint Abdulrahman University
- ☐ Qassim University
- ☐ Shaqra University
- ☐ Taibah University
- ☐ Taif University
- ☐ Umm Al-Qura University
- ☐ University of Bisha
- ☐ University of Business and Technology
- ☐ University of Ha'il
- ☐ University of Jeddah
- ☐ University of Tabuk
- ☐ Other……….

Which region in Saudi Arabia?

Do you have any type of disabilities?

- ☐ yes (will proceed to this question)
- ☐ no (will skip this question)

If you are known to have disability, please indicate your disability:

- ☐ Mobility impairments (e.g., wheelchair users)
- ☐ Visual impairments (e.g., blindness or low vision)
- ☐ Hearing impairments (e.g., deaf or hard of hearing)
- ☐ Cognitive impairments (e.g., intellectual disabilities, or learning disabilities, or Attention deficit and hyperactivity disorders))
- ☐ Autism spectrum disorders
- ☐ Other (please specify)

Section 2: Knowledge of Adaptive Sports

Directions: Read each statement below carefully. Choose a T on the line if you think a statement, it TRUE. Place an F on the line if you think the statement is FALSE.

Items	Correct Response		
1. Many of the traditional sports have an equal counterpart in adapted sports and with similar rules for individuals with disabilities			
2. Adapted sports supplemented into traditional curriculum will help in delivering a more comprehensive program and develop student interaction (i.e., social) for all skill levels			
3. Adaptive sports are sports that allow participation of all populations with inclusion of assistive devices such as wheelchairs, crutches, specially created chairs, modified hand-held equipment			
4. healthcare professionals may not make recommendations for participation in adaptive sports for individuals following a traumatic injury or onset of disease affecting mobility			
5. Adaptive sports have shown less benefits than of regular sports in community reintegration, life satisfaction, the opportunity for employment, and quality of life			
6. Individuals who are blind cannot engage in sports			

Directions: Read each question carefully, and then choose THE ANSWER that best fits the question.

1. Which of the following is not Group adaptive sports	A. Volleyball	B. Goalball	C. Basketball shoot outs
2. Which of the following is not Individual adaptive sports	A. Football penalty-shoots	B. Bowling game	C. Throwball
3. Which of the following term is used as alternative to adapted sports	A. Traditional sports	B. Parasports	C. Olympic sports
4. What is the role of physical education in adapted sports	A. To give the learner the skills necessary for a lifetime of rich leisure, recreation, and sport experiences to enhance physical fitness and wellness	B. to manipulate learner self-confidence self-esteem	C. To increase physical fitness
5. What is the two key part of Sport Framework for Individuals with Disabilities	A. sport settings and sport delivery options.	B. Type of disability and sport delivery options.	C. Type of disability and sport setting
6. Which of the following is not considered types of sport settings within the sport framework for Individuals with Disabilities	A. School or college based,	B. Home	C. community
7. What is the best way of promoting inclusion and developing a more comprehensive general physical education in sports.	A. teaching adapted sport in a general physical education curriculum	B. Establish gym for individuals with disabilities	C. Activate international days
8. What are the key steps that will assist in teaching adapted sports?	A. Determine the sport to play then cross-reference that sport to one from adapted sport	B. Assess the performance for all students on the skill of participation is not required	C. Ensure the availability of the setting
9. Why the domain of the assessment process of sport classification is established?	A. To classify sport based on impairments and performance	B. To classify sport based on the environmental barriers	C. To classify sport aased on the performance
10. How can adaptive sports contribute to improving self-esteem and social inclusion among individuals with disabilities?	A. By providing opportunities for skill development and competition.	B. By segregating athletes with disabilities from mainstream sports.	C. By excluding individuals with intellectual disabilities from participating.

Section 3: Awareness

Please answer the following question:

Questions	Response		
	Yes	No	I am not sure
Have you heard of the term "adaptive sports" before taking this survey?			
Have you ever attended an adaptive sports event or competition?			

Have you or anyone you know participated in an inclusive adaptive sports or sport activities for individuals with disabilities at the university?			
Are there currently any inclusive adaptive sports programs or initiatives offered at your university?			
Have you ever received any formal education or training, or orientation related to adaptive sports or inclusion?			
Are you familiar with the classification system used in adaptive sports to ensure fair competition among participants with varying disabilities?			

Section 4: Perception

To what extent do you agree with the following statement:

Items	Response				
	Strongly Agree	Agree	Neutral	Disagree	Strongly Disagree
The increased knowledge about adaptive sports can benefit your university by improving inclusion and participation					
The university play a significant role in promoting in-depth knowledge and understanding of adaptive sports among students and faculty.					
Inclusive adaptive sports programs can have a positive impact on the overall well-being of students with disabilities					
Parasport opportunities enhance the sense of community and belonging among students with disabilities at your university					
The existing sports facilities for students with disabilities are accessible					
There are specific policies or guidelines in place at the university to support inclusive adaptive sports programs					
There are staff or personnel responsible for coordinating inclusive adaptive sports at your university					
There is sufficient promotion of adaptive sports or sports for students with disabilities at your university					
The inclusivity of sports culture for individuals with disabilities in the university is high.					
Students with disabilities have equitable access to parasport opportunities and resources compared to their peers without disabilities					

Section 4: Strengths and Resources

Please answer the following question:

Items	Response		
	Yes	No	I am not aware
Are there specific programs or initiatives at the university gym that aim to support the participation of individuals with disabilities in sports and physical activities?			
Are there accessible and inclusive sports facilities available on campus to accommodate individuals with disabilities?			
Are there any existing resources or initiatives at the university that you believe can be leveraged to support the establishment of inclusive adaptive sports programs?			

Are there dedicated facilities or spaces within the university designed to accommodate individuals with disabilities for sports and physical activities?			
Does Parasport receive equal attention and funding as mainstream sports within our university's athletic programs?			
The university's investment in parasport facilities and resources is commensurate with the needs and interests of students with disabilities			

Section 5: Barriers to Participation

In your opinion, what are the main barriers that students with disabilities might face when participating in sports activities at your university? (Select all that apply)

- ☐ Lack of accessible facilities
- ☐ Social stigma and discrimination
- ☐ Lack of awareness and information
- ☐ Financial constraints
- ☐ Limited equipment and resources
- ☐ Other (please specify)
- ☐ I don't know

Section 6: For Students with disability

Based on your perspective, what potential obstacles might hinder the establishment of adaptive sports programs within Saudi universities?

Personal factors:

☐ Not being able to exercise because of the disability
☐ I do not like participating in sports
☐ I do not have enough energy/ I am too fatigued to participate in sports
☐ I have an injury that prevents me from participating in sports
☐ Being (too) busy with other activities
☐ Not being comfortable in the presence of other athletes
☐ I am ashamed of my disability
☐ Being dependent of others to be able to exercise
☐Other,(please specify)..

Social and environmental factors:

☐ Sports possibilities are unknown
☐ Having little sports possibilities in the neighborhood, no/not sufficiently qualified supervision
☐ Facilities not (sufficiently) adjusted
☐ Financial constraints
☐ Transportations
☐ Materials not (sufficiently) adjusted or available.
☐ Practice/Training is not (sufficiently) adapted.
☐ Lack of possibilities to exercise with peers. Disabled athletes are not (fully) accepted.
☐ Lack of fellow athletes with a disability/ sports buddy
☐ Could not find a fitting sport that fit me
☐ I don't know
☐ Other, ,(please specify)namely..

How do you think these barriers can be addressed or mitigated? (Qualitative question)?

Given the researchers' desire to investigate in depth the perceptions regarding the importance and benefits of adoptive sports, researchers wishes to conduct interviews, which will be treated with the utmost confidentiality. If you wish, please write your name and mobile number and we will contact you to arrange the interview at a time that suits you.

آراء أعضاء هيئة التدريس والطلاب ووعيهم تجاه الرياضات التكيفية الشاملة في الجامعات السعودية

أولاً: المعرفة بالرياضات التكيفية

الجزء الأول: صحيح أم خطأ

التعليمات: اقرأ كل عبارة أدناه بعناية. ضع علامة (✓) في خانة «صحيح» إذا كانت العبارة صحيحة، وفي خانة «خطأ» إذا كانت خاطئة.

العبارة	صحيح ✓	خطأ ✓
كثيرٌ من الرياضات التقليدية لها نظير مماثل في الرياضات التكيفية بقواعد مشابهة للأفراد ذوي الإعاقة.	☐	☐
إدراج الرياضات التكيفية في المناهج التقليدية سيساعد في تقديم برنامج أكثر شمولاً وتطوير التفاعل الاجتماعي بين الطلاب.	☐	☐
الرياضات التكيفية هي رياضات تتيح المشاركة لجميع الفئات بما فيها الأجهزة المساعدة كالكراسي المتحركة والعكازات والمعدات المعدّلة.	☐	☐
لا يجوز للمختصين الصحيين تقديم توصيات بالمشاركة في الرياضات التكيفية للأفراد الذين يعانون من إصابات أو أمراض تؤثر على حركتهم.	☐	☐
أظهرت الرياضات التكيفية فوائد أقل مقارنةً بالرياضات الاعتيادية في الاندماج المجتمعي والرضا عن الحياة والتوظيف والجودة العامة.	☐	☐
لا يستطيع الأفراد المكفوفون ممارسة أي رياضة.	☐	☐

الجزء الثاني: اختر الإجابة الصحيحة

التعليمات: اقرأ كل سؤال بعناية، ثم اختر الإجابة الأنسب من بين الخيارات المتاحة.

السؤال	أ	ب	ج
**1. أيٌّ مما يلي ليس من الرياضات التكيفية الجماعية؟**	أ. الكرة الطائرة	ب. الغولبول	ج. تسديدات السلة
**2. أيٌّ مما يلي ليس من الرياضات التكيفية الفردية؟**	أ. ركلات جزاء كرة القدم	ب. لعبة البولينغ	ج. كرة الرمي
**3. أيٌّ من المصطلحات التالية يُستخدم بديلاً عن الرياضات التكيفية؟**	أ. الرياضات التقليدية	ب. رياضات البارا	ج. الألعاب الأولمبية
**4. ما هو دور التربية البدنية في الرياضات التكيفية؟**	أ. تزويد المتعلم بالمهارات اللازمة لحياة غنية بالترفيه والاستجمام والرياضة لتعزيز اللياقة والصحة	ب. التأثير في ثقة المتعلم بنفسه واحترامه لذاته	ج. تحسين اللياقة البدنية فقط

السؤال	أ	ب	ج
**5. ما هو الجزءان الأساسيان في إطار عمل الرياضة للأفراد ذوي الإعاقة؟**	أ. بيئات الرياضة وخيارات تقديمها	ب. نوع الإعاقة وخيارات تقديم الرياضة	ج. نوع الإعاقة والبيئة الرياضية
**6. أيٌّ مما يلي لا يُعتبر من أنواع البيئات الرياضية في إطار عمل رياضة الأفراد ذوي الإعاقة؟**	أ. المدرسة أو الكلية	ب. المنزل	ج. المجتمع
**7. ما هي أفضل طريقة لتعزيز الاندماج وتطوير تربية بدنية عامة أكثر شمولاً؟**	أ. تدريس الرياضة التكيفية في مناهج التربية البدنية العامة	ب. إنشاء صالة رياضية مخصصة للأفراد ذوي الإعاقة	ج. تفعيل الأيام الدولية للتوعية
**8. ما هي الخطوات الرئيسية التي تساعد في تدريس الرياضات التكيفية؟**	أ. تحديد الرياضة المراد ممارستها ثم مقارنتها بنظيرتها في الرياضات التكيفية	ب. تقييم أداء جميع الطلاب في مهارة المشاركة دون اشتراط ذلك	ج. ضمان توافر البيئة المناسبة
**9. لماذا تم إنشاء مجال عملية التقييم في تصنيف الرياضة؟**	أ. لتصنيف الرياضة بناءً على الإعاقات والأداء	ب. لتصنيف الرياضة بناءً على العوائق البيئية	ج. لتصنيف الرياضة بناءً على مستوى الأداء فقط
**10. كيف تُسهم الرياضات التكيفية في تحسين تقدير الذات والاندماج الاجتماعي لدى الأفراد ذوي الإعاقة؟**	أ. من خلال توفير فرص لتطوير المهارات والتنافس	ب. من خلال عزل الرياضيين ذوي الإعاقة عن الرياضات السائدة	ج. من خلال استبعاد الأفراد ذوي الإعاقات الذهنية من المشاركة

ثانياً: قسم الوعي تجاه الرياضة التكيفية

التعليمات: أختر الإجابة الملائمة

السؤال	نعم	لا	لست متأكداً
هل سبق لك أن سمعت بمصطلح «الرياضات التكيفية» قبل هذا الاستبيان؟	☐	☐	☐
هل سبق لك حضور فعالية أو منافسة رياضية تكيفية؟	☐	☐	☐
هل سبق لك أو لأي شخص تعرفه المشاركة في رياضات تكيفية شاملة أو أنشطة رياضية للأفراد ذوي الإعاقة في الجامعة؟	☐	☐	☐
هل توجد حالياً أي برامج أو مبادرات للرياضات التكيفية الشاملة في جامعتك؟	☐	☐	☐
هل تلقّيت أي تعليم رسمي أو تدريباً أو توجيهاً يتعلق بالرياضات التكيفية أو الاندماج؟	☐	☐	☐
هل أنت على دراية بنظام التصنيف المستخدم في الرياضات التكيفية لضمان المنافسة العادلة بين المشاركين ذوي الإعاقات المختلفة؟	☐	☐	☐

ثالثاً: قسم الاتجاهات أووجهة النظر تجاه الرياضة التكيفية

التعليمات: حدّد مدى موافقتك على كل عبارة من العبارات التالية بوضع علامة (✓) في الخانة المناسبة.

العبارة	أو افق بشدة	أو افق	محايد	لا أو افق	لا أو افق بشدة
زيادة المعرفة بالرياضات التكيفية يمكن أن تفيد جامعتك من خلال تحسين الاندماج والمشاركة	☐	☐	☐	☐	☐
تضطلع الجامعة بدور مهم في تعزيز المعرفة العميقة وفهم الرياضات التكيفية بين الطلاب وأعضاء هيئة التدريس	☐	☐	☐	☐	☐
يمكن لبرامج الرياضات التكيفية الشاملة أن يكون لها أثر إيجابي على الرفاه العام للطلاب ذوي الإعاقة	☐	☐	☐	☐	☐
تُعزز فرص رياضات البارا الشعور بالانتماء والمجتمع لدى الطلاب ذوي الإعاقة في جامعتك	☐	☐	☐	☐	☐
المرافق الرياضية الحالية للطلاب ذوي الإعاقة ميسَّرة وسهلة الوصول	☐	☐	☐	☐	☐
توجد سياسات أو مبادئ توجيهية محددة في الجامعة لدعم برامج الرياضات التكيفية الشاملة	☐	☐	☐	☐	☐
يوجد موظفون أو كوادر مسؤولون عن تنسيق الرياضات التكيفية الشاملة في جامعتك	☐	☐	☐	☐	☐
يوجد ترويج كافٍ للرياضات التكيفية أو رياضات الطلاب ذوي الإعاقة في جامعتك	☐	☐	☐	☐	☐
ثقافة الرياضة الشاملة للأفراد ذوي الإعاقة في الجامعة عالية المستوى	☐	☐	☐	☐	☐
يتمتع الطلاب ذوو الإعاقة بإمكانية وصول متساوية لفرص رياضات البارا والموارد مقارنةً بأقرانهم	☐	☐	☐	☐	☐

رابعاً: قسم نقاط القوة والموارد

التعليمات: أجب عن الأسئلة التالية بوضع علامة (✓) في الخانة المناسبة.

السؤال	نعم	لا	لست على علم
هل توجد برامج أو مبادرات محددة في الصالة الرياضية بالجامعة تهدف إلى دعم مشاركة الأفراد ذوي الإعاقة في الرياضة والأنشطة البدنية؟	☐	☐	☐
هل تتوفر مرافق رياضية ميسَّرة وشاملة في الحرم الجامعي لاستيعاب الأفراد ذوي الإعاقة؟	☐	☐	☐
هل توجد موارد أو مبادرات قائمة في الجامعة يمكن الاستفادة منها لدعم إنشاء برامج رياضات تكيفية شاملة؟	☐	☐	☐
هل توجد مرافق أو مساحات مخصصة داخل الجامعة مصممة لاستيعاب الأفراد ذوي الإعاقة في الرياضة والأنشطة البدنية؟	☐	☐	☐
هل تحظى رياضات البارا باهتمام وتمويل مساوٍ للرياضات الرئيسية ضمن البرامج الرياضية في جامعتك؟	☐	☐	☐

خامساً: العوائق أمام المشاركة

في رأيك، ما هي العوائق الرئيسية التي قد يواجهها الطلاب ذوو الإعاقة عند المشاركة في الأنشطة الرياضية في جامعتك؟

(يمكنك اختيار أكثر من إجابة)

العائق	اختر ✓
غياب المرافق الميسَّرة وسهلة الوصول	☐
الوصمة الاجتماعية والتمييز	☐
ضعف الوعي ونقص المعلومات	☐
القيود المالية	☐
محدودية المعدات والموارد	☐
لا أعرف	☐
أخرى، يرجى التحديد:..	☐

سادساً: قسم الطلاب ذوي الإعاقة

بناءً على منظورك، ما هي العقبات المحتملة التي قد تحول دون إنشاء برامج رياضية تكيفية داخل الجامعات السعودية؟

أولاً: العوامل الشخصية

☐عدم القدرة على ممارسة الرياضة بسبب الإعاقة

☐لا أرغب في المشاركة في الأنشطة الرياضية

☐لا تتوفر لديّ طاقة كافية / أشعر بالإرهاق الشديد الذي يحول دون ممارسة الرياضة

☐لديّ إصابة تمنعني من المشاركة في الأنشطة الرياضية

☐الانشغال بأنشطة أخرى

☐الشعور بعدم الارتياح في حضور الرياضيين الآخرين

☐الشعور بالخجل من إعاقتي

☐الاعتماد على الآخرين لممارسة الرياضة

☐أخرى، يرجى التحديد: ....

ثانياً: العوامل الاجتماعية والبيئية

☐عدم الإلمام بالفرص الرياضية المتاحة

☐شُح الفرص الرياضية في المنطقة المحيطة، أو غياب إشراف مؤهَّل أو عدم كفايته

☐المرافق غير مهيَّأة أو غير مكيَّفة بصورة كافية

☐القيود المالية

☐صعوبات التنقل والمواصلات

☐المعدات غير مكيَّفة أو غير متاحة بصورة كافية

☐التدريب والممارسة غير مكيَّفَين بصورة كافية

☐ضعف فرص ممارسة الرياضة مع الأقران — الرياضيون ذوو الإعاقة لا يحظون بقبول كامل

☐غياب رياضيين آخرين من ذوي الإعاقة أو رفيق رياضي مناسب

☐عدم العثور على رياضة تلائمني وتناسب قدراتي

☐لا أعرف

☐أخرى، يرجى التحديد: ....

سابعاً: سؤال مفتوح

كيف تعتقد أنه يمكن معالجة هذه العوائق أو التخفيف منها؟

ثامناً: الرغبة في المشاركة بالمقابلة

رغبةً من فريق البحث في التعمق في استيعاب التصورات المتعلقة بأهمية الرياضات التكيفية وفوائدها وتداعياتها، يسعى الباحثون إلى إجراء مقابلات فردية معمّقة ستُعامَل بأقصى درجات السرية والخصوصية التامة.

إذا كنت ترغب في المشاركة، يُرجى كتابة اسمك ورقم جوالك أدناه وسنتواصل معك لترتيب المقابلة في الوقت الذي يناسبك.
