# Supplementary material for: Advancing inclusion in sports for students with disability: A mixed-methods study on awareness and perspectives toward adaptive sports
Source: PLoS One. 2026 May 20;21(5):e0349033. doi: 10.1371/journal.pone.0349033 (PMC13189293; doi:10.1371/journal.pone.0349033)
Supplement: S3 File — (PDF) [file pone.0349033.s003.pdf]

## Supporting information 3:Reliability Analysis and Factor Analysis of the questionnaire

### Reliability Analysis

Prior to conducting the factor analyses, the internal consistency of the three measurement scales was assessed using Cronbach's alpha (α). Results indicated satisfactory to excellent levels of reliability across all three instruments. The 16-item Knowledge scale , yielded a reliability coefficient of α = .878, indicating good internal consistency. The six-item awareness scale which also included a reversed item (Item1), demonstrated acceptable reliability (α = .758). The 10-item Likert-format perception scale exhibited excellent internal consistency (α = .945). A summary of the reliability statistics is presented in Table 1.

*Table 1*. Summary of Reliability Statistics for the Three Measurement Scales

| **Scale** | **Items (k)** | **Avg. Interitem Covariance** | **Cronbach's α** |
|---|---|---|---|
| Knowledge | 16 | .068 | .878 |
| Awareness | 6 | .069 | .758 |
| Perception | 10 | .997 | .945 |

*Note.* n = 30. Scale reliability estimated using Cronbach's alpha. Avg. = average; k = number of items in scale.

### Factor Analysis

Principal component factor analyses were conducted separately for each scale. Factors with eigenvalues exceeding 1.0 (Kaiser criterion) were retained. All analyses were based on n = 30 observations.

**Knowledge Scale.** Factor analysis of the 16-item Knowledge scale yielded six factors with eigenvalues greater than 1.0, collectively accounting for 80.78% of the total variance. Factor 1 was the dominant component, explaining 38.43% of the variance (eigenvalue = 6.149). The remaining factors explained successively smaller proportions: Factor 2 (13.27%, eigenvalue = 2.124), Factor 3 (8.17%, eigenvalue = 1.307), Factor 4 (7.41%, eigenvalue = 1.186), Factor 5 (6.94%, eigenvalue = 1.110), and Factor 6 (6.57%, eigenvalue = 1.051). The likelihood ratio test confirmed that the factor structure differed significantly from the independence model, $\chi^2(120) = 282.00$, $p < .001$. Factor loadings and unique variances are presented in Table 2.

*Table 2. Factor Loadings and Unique Variances for the 16-Item Knowledge Scale*

| **Variable** | **Factor Loading** | | | | | | **Uniqueness** |
|---|---|---|---|---|---|---|---|
| | *F1* | *F2* | *F3* | *F4* | *F5* | *F6* | |
| Item 1 | −.619 | .500 | .130 | .250 | .225 | .107 | .226 |
| Item 2 | .817 | .082 | −.131 | −.295 | −.340 | −.104 | .096 |
| Item 3 | .761 | −.142 | −.024 | −.163 | −.317 | .208 | .231 |
| Item 4 | .062 | −.320 | .786 | −.228 | −.222 | .055 | .172 |
| Item 5 | .482 | .561 | −.043 | .158 | −.110 | −.481 | .183 |
| Item 6 | .797 | .065 | .085 | −.061 | −.304 | .176 | .227 |

| Variable | Factor Loading | | | | | | Uniqueness |
|---|---|---|---|---|---|---|---|
| | *F1* | *F2* | *F3* | *F4* | *F5* | *F6* | |
| Item 7 | .612 | −.009 | .170 | −.278 | .318 | .125 | .402 |
| Item 8 | .394 | −.645 | −.113 | .008 | .080 | −.495 | .166 |
| Item 9 | .732 | .427 | −.233 | .164 | −.161 | .006 | .175 |
| Item 10 | .859 | .040 | −.153 | .154 | .095 | −.165 | .177 |
| Item 11 | .281 | −.528 | .081 | .724 | −.132 | .118 | .081 |
| Item 12 | .476 | −.120 | −.467 | −.144 | .278 | .566 | .123 |
| Item 13 | .537 | −.529 | −.081 | .172 | .355 | −.062 | .266 |
| Item 14 | .673 | .013 | .363 | −.085 | .419 | −.087 | .225 |
| Item 15 | .622 | .274 | .340 | .466 | −.059 | .285 | .121 |
| Item 16 | .641 | .422 | .220 | −.060 | .384 | −.080 | .206 |
| *Eigenvalue* | 6.149 | 2.124 | 1.307 | 1.186 | 1.110 | 1.051 | |
| *% Variance* | 38.43 | 13.27 | 8.17 | 7.41 | 6.94 | 6.57 | |
| *Cumulative %* | 38.43 | 51.70 | 59.87 | 67.28 | 74.21 | 80.78 | |

*Note.* n = 30. Method: principal component factors; rotation: unrotated. F = Factor. RSuperscript R indicates reversed item. LR test for independence vs. saturated model: $\chi^2(120) = 282.00$, $p < .001$.

**Awareness Scale.** Factor analysis of the six-item Attitude scale retained two factors with eigenvalues exceeding 1.0, together accounting for 69.17% of the total variance. Factor 1 explained the largest share of variance (49.50%, eigenvalue = 2.970), while Factor 2 contributed an additional 19.67% (eigenvalue = 1.180). The model fit was significant, $\chi^2(15) = 63.14$, $p < .001$. Factor loadings and uniqueness values are reported in Table 3.

*Table 3*
*Factor Loadings and Unique Variances for the 6-Item Attitude Scale*

| Variable | Factor 1 | Factor 2 | Uniqueness |
|---|---|---|---|
| Item 1 | −.711 | .233 | .440 |
| Item 2 | .888 | .139 | .192 |
| Item 3 | .845 | −.084 | .278 |
| Item 4 | .184 | −.824 | .287 |
| Item 5 | .490 | .645 | .343 |
| Item 6 | .829 | −.063 | .309 |
| *Eigenvalue* | 2.970 | 1.180 | |
| *% Variance* | 49.50 | 19.67 | |
| *Cumulative %* | 49.50 | 69.17 | |

*Note.* n = 30. Method: principal component factors; rotation: unrotated. RSuperscript R indicates reversed item. LR test for independence vs. saturated model: $\chi^2(15) = 63.14$, $p < .001$.

**Perception Scale.** Factor analysis of the 10-item Likert-format scale produced two factors with eigenvalues greater than 1.0, explaining a combined 84.65% of the total variance. Factor 1 was dominant, accounting for 67.43% of the variance (eigenvalue = 6.743), while Factor 2 explained an additional 17.22% (eigenvalue = 1.722). The LR test confirmed statistical significance, $\chi^2(45) = 408.08$, $p < .001$. All items loaded strongly on Factor 1 (loadings ranging from .684 to .918), with items 1 through 4 showing positive secondary loadings on Factor 2 and items 5 through A10 showing negative secondary loadings, suggesting a potential subdimensional structure. Detailed factor loadings are presented in Table 4.

*Table 4*
*Factor Loadings and Unique Variances for the 10-Item Likert Attitude–Practice Scale*

| **Variable** | **Factor 1** | **Factor 2** | **Uniqueness** |
|---|---|---|---|
| Item 1 | .684 | .525 | .257 |
| Item 2 | .739 | .516 | .187 |
| Item 3 | .793 | .539 | .080 |
| Item 4 | .812 | .532 | .058 |
| Item 5 | .872 | −.312 | .141 |
| Item 6 | .874 | −.239 | .180 |
| Item 7 | .918 | −.316 | .058 |
| Item 8 | .779 | −.419 | .218 |
| Item 9 | .886 | −.255 | .151 |
| Item 10 | .827 | −.334 | .205 |
| *Eigenvalue* | 6.743 | 1.722 | |
| *% Variance* | 67.43 | 17.22 | |
| *Cumulative %* | 67.43 | 84.65 | |

*Note.* n = 30. Method: principal component factors; rotation: unrotated. LR test for independence vs. saturated model: $\chi^2(45) = 408.08$, $p < .001$.
