# Supplementary material for: Advancing inclusion in sports for students with disability: A mixed-methods study on awareness and perspectives toward adaptive sports
Source: PLoS One. 2026 May 20;21(5):e0349033. doi: 10.1371/journal.pone.0349033 (PMC13189293; doi:10.1371/journal.pone.0349033)
Supplement: S4 File — (PDF) [file pone.0349033.s004.pdf]

Checklist for Reporting Results of Internet E-Surveys (CHERRIES)

Checklist Item	***Explanation***	***Page Number***
Describe survey design	Explanatory sequential mixed-methods; cross-sectional online survey using SurveyMonkey; convenience sampling	p.5–7
IRB approval	Institutional Review Board approval (HAP-01-R-059; IRB Log: 23-0876)	p.6
Informed consent	Electronic consent via survey cover page; included purpose, confidentiality, voluntary participation, withdrawal rights	p.6
Data protection	Data stored on password-protected computer; anonymity ensured via pseudonyms (qualitative)	P.10
Development and testing	Questionnaire developed via literature review + expert panel + pilot study (n=30); reliability & validity assessed	p.8–10
Open survey versus closed survey	Closed survey (distributed via university mailing lists and networks)	p.6
Contact mode	Online recruitment via email, university platforms	p.6
Advertising the survey	Distributed through university mailing lists, student platforms, academic networks; reminders every 2 weeks	p.6
Web/E-mail	Web-based survey (SurveyMonkey platform)	p.8
Context	Conducted across Saudi universities (students, faculty, staff)	p.6–7
Mandatory/voluntary	Voluntary participation	p.6
Incentives	No incentives reported	NA
Time/Date	Jan 1 – Aug 31, 2024 (quantitative phase)	p.6
Randomization of items or questionnaires	Not reported	NA
Adaptive questioning	Not reported	NA
Number of Items	6 sections; knowledge (16 items), awareness (6), perception (10), others	p.8–9
Number of screens (pages)	Not reported	NA
Completeness check	Incomplete responses excluded from analysis	p.12
Review step	NA	NA
Unique site visitor	Not reported	NA
View rate (Ratio of unique survey visitors/unique site visitors)	Not reported	NA

Participation rate (Ratio of unique visitors who agreed to participate/unique first survey page visitors)	Not explicitly reported	NA
Completion rate (Ratio of users who finished the survey/users who agreed to participate)	410 responses → 350 analyzed after exclusions	p.12
Cookies used	Not reported	NA
IP check	Not reported	NA
Log file analysis	Not reported	NA
Registration	Not reported	NA
Handling of incomplete questionnaires	60 responses excluded due to missing/ineligible data	p.12
Questionnaires submitted with an atypical timestamp	Not reported	NA
Statistical correction	No weighting; descriptive statistics used	p.11

This checklist has been modified from Eysenbach G. Improving the quality of Web surveys: the Checklist for Reporting Results of Internet E-Surveys (CHERRIES). J Med Internet Res. 2004 Sep 29;6(3):e34 [erratum in J Med Internet Res. 2012; 14(1): e8.]. Article available at https://www.jmir.org/2004/3/e34/; erratum available https://www.jmir.org/2012/1/e8/. Copyright ©Gunther Eysenbach. Originally published in the Journal of Medical Internet Research, 29.9.2004 and 04.01.2012.

This is an open-access article distributed under the terms of the Creative Commons Attribution License (https://creativecommons.org/licenses/by/2.0/), which permits unrestricted use, distribution, and reproduction in any medium, provided the original work, first published in the Journal of Medical Internet Research, is properly cited.
